# More Is Not Always Better—the Double-Headed Role of Fibronectin in Staphylococcus aureus Host Cell Invasion

**DOI:** 10.1128/mBio.01062-21

**Published:** 2021-10-19

**Authors:** Silke Niemann, Minh-Thu Nguyen, Johannes A. Eble, Achmet I. Chasan, Maria Mrakovcic, Ralph T. Böttcher, Klaus T. Preissner, Steffen Roßlenbroich, Georg Peters, Mathias Herrmann

**Affiliations:** a Institute of Medical Microbiology, University Hospital Münster, Münster, Germany; b Institute of Physiological Chemistry and Pathobiochemistry, University of Münster, Münster, Germany; c Institute of Immunology, University of Münster, Münster, Germany; d Department of Molecular Medicine, Max Planck Institute for Biochemistry, Martinsried, Germany; e Kerckhoff-Herzforschungsinstitut, Department of Cardiology, Medical School, Justus-Liebig-University, Giessen, Germany; f Department of Trauma, Hand and Reconstructive Surgery, University Hospital Münster, Münster, Germany; g Institute of Infectiology, University of Münster, Münster, Germany; University of Rochester

**Keywords:** *Staphylococcus*, epithelial cells, fibronectin, host cell invasion, host-pathogen interactions, osteoblasts

## Abstract

While Staphylococcus aureus has classically been considered an extracellular pathogen, these bacteria are also capable of being taken up by host cells, including nonprofessional phagocytes such as endothelial cells, epithelial cells, or osteoblasts. The intracellular S. aureus lifestyle contributes to infection development. The predominant recognition and internalization pathway appears to be the binding of the bacteria via a fibronectin bridge to the α5β1-integrin on the host cell membrane, followed by phagocytosis. Although osteoblasts showed high expression of α5β1-integrin and fibronectin, and bacteria adhered to osteoblasts to a high proportion, here we demonstrate by internalization assays and immunofluorescence microscopy that S. aureus was less engulfed in osteoblasts than in epithelial cells. The addition of exogenous fibronectin during the infection of cells with S. aureus resulted in an increased uptake by epithelial cells but not by osteoblasts. This contrasts with the previous conception of the uptake mechanism, where high expression of integrin and fibronectin would promote the bacterial uptake into host cells. Extracellular fibronectin surrounding osteoblasts, but not epithelial cells, is organized in a fibrillary network. The inhibition of fibril formation, the short interfering RNA-mediated reduction of fibronectin expression, and the disruption of the fibronectin-fibril meshwork all resulted in a significant increase in S. aureus uptake by osteoblasts. Thus, the network of fibronectin fibrils appears to strongly reduce the uptake of S. aureus into a given host cell, indicating that the supramolecular structure of fibronectin determines the capacity of particular host cells to internalize the pathogen.

## INTRODUCTION

Traditionally, Staphylococcus aureus has been considered a pyogenic extracellular pathogen, based on the typical histopathologic feature of an invasive S. aureus infection, i.e., the typical presence of microorganisms in the extracellular spaces of diseased tissue and the subsequent formation of an abscess. However, since the 2000s, the renewed assessment of clinical hallmarks in S. aureus*-*mediated infections, such as the predilection of endovascular infection sites, the frequent failure of antimicrobial therapy, and the ability of the pathogen to cause relapsing disease (in some instances even decades after the initial infection), has firmly established the concept of eukaryotic invasion by the pathogen. This not only has been proven to be important for our understanding of the pathogenesis of S. aureus (1) but also resulted in shifts in clinical practice, such as extended duration of antimicrobial therapy. Meanwhile, this concept is supported by the demonstration of S. aureus in endovascular, bone, and soft tissue cells in experimental infection as well as in authentic clinical human colonization and disease (2–10).

The mechanisms of staphylococcal uptake by eukaryotes have been elucidated in appreciable detail. In contrast to uptake mechanisms, e.g., of enterobacteriaceae that require active participation of the microorganisms by various secretion systems, even inactivated S. aureus microbes are phagocytosed by host cells, in most instances with the help of bridging molecules. Fibronectin (Fn) was the first adhesive protein implicated in such bridge formation spanning between the Fn binding proteins (FnBP) A and B on the staphylococcal surface and the α5β1 integrin on the eukaryotic cell surface (11, 12).

More recently, it has been observed that Fn-independent bacterial uptake mechanisms may promote cellular invasion either in concert with the classical Fn-mediated phagocytosis or even in the absence of Fn and/or its ligands. Some examples of these “alternative” uptake mechanisms include the iron-regulated surface determinant B (IsdB) protein that may serve as a bacterial receptor for vitronectin, which itself binds to αvβ3 integrin on the host cell (13). Moreover, the interaction of S. aureus autolysin Atl with heat shock cognate protein Hsc70 promotes the internalization of S. aureus (14, 15), whereas the bacterial extracellular adherence protein (Eap) mediates cellular uptake, possibly by its binding to a variety of host proteins (16, 17). S. aureus lipoproteins also have been shown to trigger bacterial uptake into host cells by activating heat shock protein 90 (18, 19).

The Fn-mediated uptake mechanism hitherto remains a prominent, efficient, and clinically relevant pathogenicity trait of S. aureus. Hence, S. aureus binds to host cell α5β1 integrin via a fibronectin bridge with the help of S. aureus FnBPs. Since one FnBP can bind 6 to 9 Fn molecules, clustering of the α5β1 integrins can occur on the host cell side, which in turn initiates intracellular signaling cascades, involving FAK and Src signaling pathways that ultimately lead to a reorganization of the actin cytoskeleton and the mobilization of the endocytosis machinery (20). This has been demonstrated for a variety of cells, including endothelial cells, the kidney cell line 293T, and, lately, osteoblasts (4, 21, 22).

Recently, our group has investigated the uptake of different S. aureus strains in various host cell types as well as the respective postinvasion events (23). Intriguingly, we found that the bacterial uptake is strongly dependent on host cell type. Primary human vein endothelial cells (HUVECs) as well as endothelial and epithelial cell lines (EA.hy926 and A549 cells) demonstrated a highly efficient uptake. In contrast, following exposure to microbes, primary human osteoblasts (pHOB) or a fibroblast cell line (CCD-32SK) exhibited a much smaller number of internalized bacteria. This observation, which is not explainable by an apparent lack of Fn or of Fn ligands on either the cellular or bacterial side, prompted us to investigate the mechanisms underlying this profound difference in uptake efficacy in more detail. In the present work we have focused on A549 and pHOB cells to elucidate their diverse bacterial uptake efficacy with regard to the expression level and fibrillary assembly of extracellular Fn. Results indicate that a fibrillary assembly of Fn on cells prevents an efficient uptake of bacteria and may provide protection of host cells against the intracellular expansion of bacteria.

## RESULTS

### Comparison of staphylococcal uptake by A549 epithelial cells and pHOBs.

**FIG 1:**
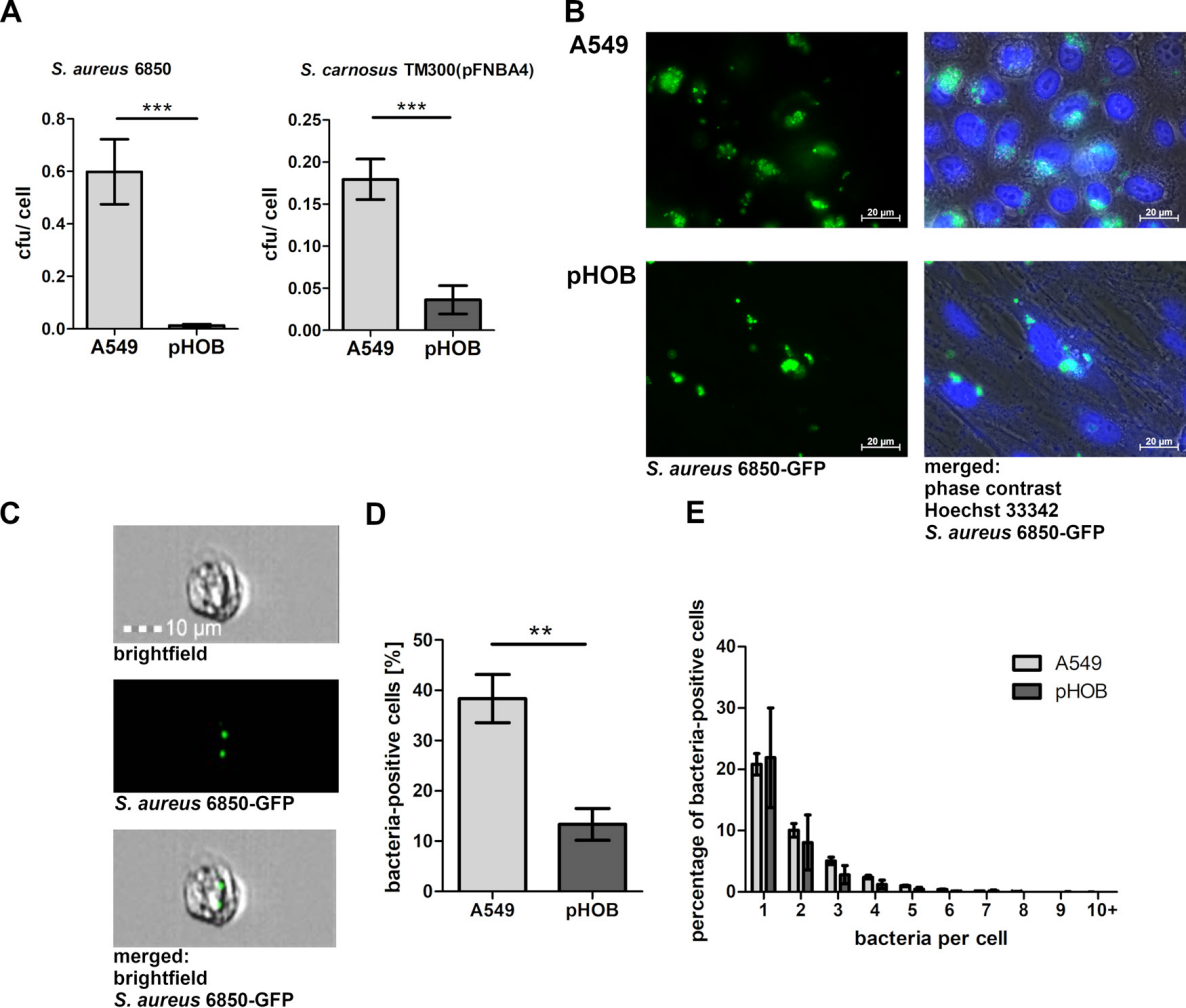
Staphylococci are taken up by A549 epithelial cells to a greater extent than by primary human osteoblasts. (A) A549 epithelial cells and pHOBs were infected with either S. aureus strain 6850 or *S. carnosus* TM300(pFnBA4) (MOI 50). One hour post infection, extracellular staphylococci were removed by lysostaphin treatment. Subsequently, the host cells were detached and the number of host cells was determined. Host cells were lysed and number of viable intracellular bacteria was assessed by plate counting. Data represent the means ± SD from four independent experiments. ***, *P < *0.001, unpaired *t* test. (B) Representative fluorescence microscopy images of A549 cells or pHOBs infected with S. aureus 6850-GFP for 1 h, followed by lysostaphin incubation and fixation (green, S. aureus 6859-GFP; blue, Hoechst 33342 nucleic acid stain). (C) Imaging flow-cytometric analysis allows microscopic visualization of analyzed cells. Representative images of an A549 cell with two GFP fluorescent spots (green). (D and E) Imaging flow-cytometric analysis of A549 cells and pHOBs 1 h post infection with S. aureus 6850-GFP, lysostaphin step, and fixation. (D) Analysis of bacterium-positive cells. Data represent the means ± SD from three independent experiments. **, *P ≤ *0.01, unpaired *t* test. (E) Spot count analysis. Data represent the means ± SD from three independent experiments, two-way ANOVA followed by Bonferroni posttests.

To compare the invasion of S. aureus into A549 epithelial cells and pHOBs, either S. aureus 6850 (a clinical strain originally recovered from a patient with osteomyelitis [24, 25]) or *S. carnosus* TM300(pFNBA4) (an environmental strain used for meat processing, engineered to express FnBPA [26]) was used to infect both cell types, carried out by the well-established lysostaphin protection assay. Both bacterial strains were taken up to a significantly larger extent by A549 cells than by pHOBs (Fig. 1A). Compared to pHOB cells, the uptake of S. aureus 6850 by A549 cells was more than 20-fold increased, whereas this increase was less pronounced using *S. carnosus* transformant. Fluorescence microscopy images (Fig. 1B) revealed that not all cells demonstrated bacterial engulfment (S. aureus 6850-GFP) in the confluent layers of large pHOBs or the smaller A549 epithelial cells. To quantify the percentage of cells containing internalized bacteria as well as the bacterial load in these cells, imaging flow cytometry analysis was employed. This method combines high-throughput flow-cytometric analysis with the imaging capacity of fluorescence microscopy (Fig. 1C). As shown in Fig. 1D, the proportion of cells containing bacteria was found to be significantly larger in A549 than pHOB cells, whereas the number of bacteria per cell did not differ significantly between the cell types (Fig. 1E).

### Contribution of α5ß1-integrin and cell-surface-associated fibronectin to bacterial uptake in A549 and pHOB cells.

**FIG 2:**
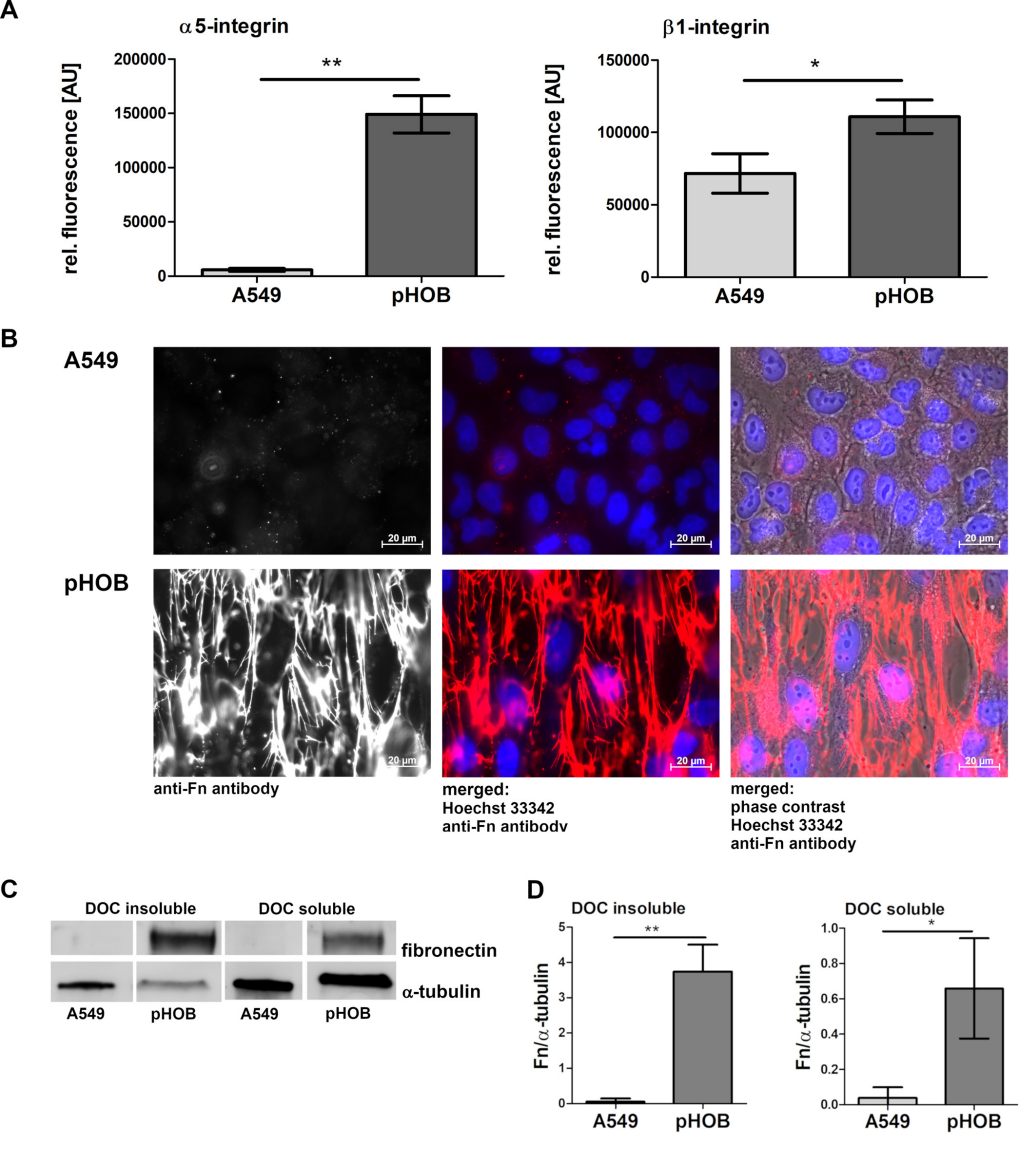
Primary osteoblasts express more α5β1-integrin than A549 cells, and the fibrillary presentation of detergent-insoluble Fn represents the prominent Fn conformation on pHOB. (A) Cells were detached with accutase and incubated with anti-α5 or anti-β1 antibody before flow-cytometric analysis. Data represent the means ± SD from 3 independent experiments. *, *P < *0.05; **, *P ≤ *0.01, unpaired *t* test. (B) Representative immunofluorescence microscopy images of Fn expression on A549 and pHOB using an antibody against Fn (white or red) and Hoechst 33342 for nucleic acid staining (blue). (C) After 3 days of growth, the DOC-insoluble and the DOC-soluble protein fractions of pHOB and A549 cells were harvested and analyzed by Western blotting. (D) Data are means ± SD from Western blot quantification of three independent experiments. *, *P < *0.05; **, *P ≤ *0.01, unpaired *t* test.

To further elucidate the differences for the bacterial uptake by both cell types, the expression of α5 and β1-integrin was compared using flow cytometry (Fig. 2A). A549 epithelial cells expressed particularly less α5 integrin and slightly less β1 integrin than the pHOB cells, clearly demonstrating a lower amount of expression of α5β1 integrin on the surface of these A549 cells. Of note, only detached cells were analyzed by flow cytometry, ruling out any inaccessibility of integrin on the basolateral versus apical cell face. In line with the elevated expression of Fn-binding α5β1integrin in pHOB cells, a prominent and dense network of fibronectin fibrils covering the surface of pHOBs was observed by immune fluorescence microscopy (Fig. 2B, IgG control, Fig. S1). In contrast, A549 cells only displayed a relatively low binding signal of anti-Fn antibody.

10.1128/mBio.01062-21.1 FIG S1 Primary osteoblast express more Fn on the cell surface as compared to A549 cells. Representative images of Fn expression on A549 and pHOB cells visualized by immunofluorescence microscopy using an antibody against Fn or IgG control antibody (red) and Hoechst 33342 for nucleic acid staining (blue). Download FIG S1, PDF file, 0.3 MB. Copyright © 2021 Niemann et al. 2021 Niemann et al. https://creativecommons.org/licenses/by/4.0/ This content is distributed under the terms of the Creative Commons Attribution 4.0 International license.

### Association of pHOBs with specific structural entities of fibronectin.

Cell-associated Fn is diffusely distributed over the surface of a tissue cell. As assembly proceeds, dimeric Fn forms short deoxycholate (DOC)-soluble fibrils that are then converted into a dense detergent-insoluble fibrillar meshwork (27) covering the surface area of cells. Accordingly, the amount of DOC-soluble and DOC-insoluble Fn was analyzed in homogenates of A549 and pHOB cells by Western blotting. While a prominent protein band was noted both for DOC-soluble and DOC-insoluble Fn in pHOB cells, almost no signals were seen in A549 cells (Fig. 2C and D, exemplary unprocessed blot in Fig. S2), indicating the existence of extensive Fn fibrils on pHOBs but not on A549 cells.

10.1128/mBio.01062-21.2 FIG S2 Representative unprocessed Western blot of DOC solubility assay of homogenates of pHOB, A549, EA.hy926, and CCD-32Sk. DOC-insoluble and DOC-soluble fractions of cell homogenates were separated by SDS-PAGE under reduced conditions and analyzed by Western blotting. In the lane labeled “Fn,” soluble Fn was applied which was used as control. The same blot is shown twice. The upper image shows the bands after staining with anti-α-tubulin antibody (loading control). Slight nonspecific bands almost at the position of the fibronectin can be detected. The lower panel additionally shows the Fn bands after anti-Fn antibody staining. This is the original Western blot on which Fig. 2C and Fig. S7A are based. Download FIG S2, PDF file, 0.1 MB. Copyright © 2021 Niemann et al. 2021 Niemann et al. https://creativecommons.org/licenses/by/4.0/ This content is distributed under the terms of the Creative Commons Attribution 4.0 International license.

These findings indicate that despite a much higher expression of Fn binding α5ß1-integrin on pHOB cells (as a prerequisite for bacterial uptake), an appreciable immobilized surface-bound Fn meshwork/fibrils appears to prevent efficient ingestion of bacteria compared to the uptake efficacy of A549 cells.

### The influence of surface-bound and soluble fibronectin on bacterial uptake by pHOBs.

**FIG 3:**
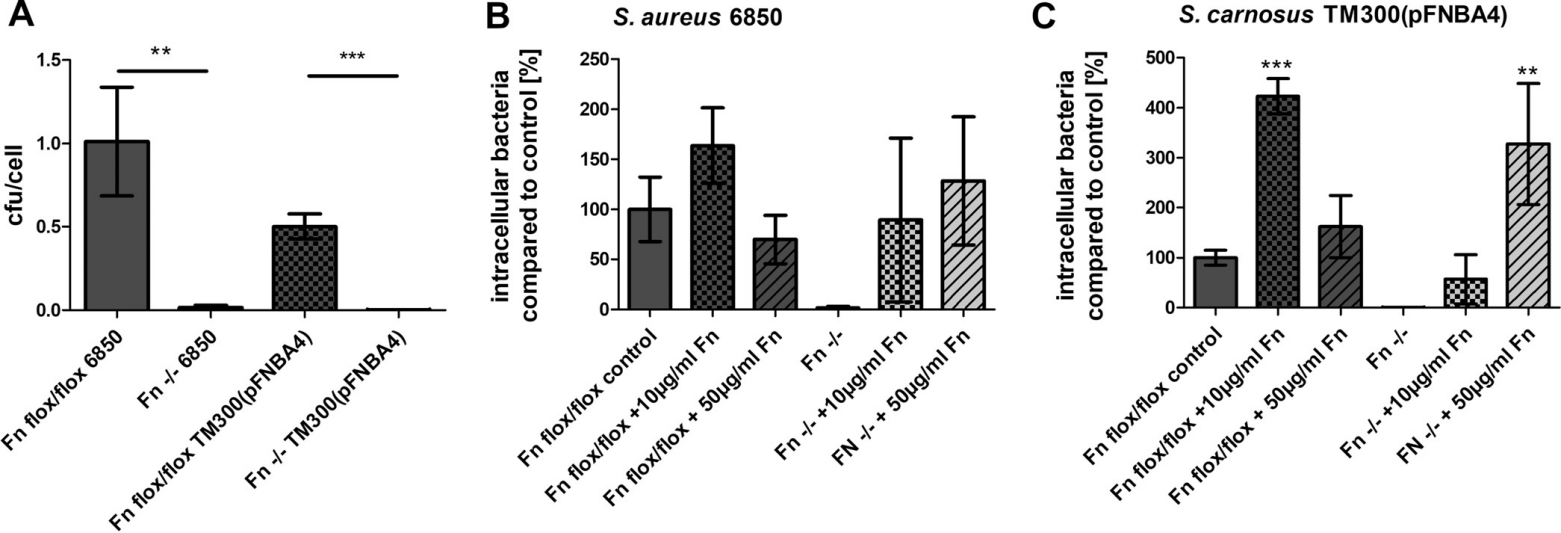
Bacterial uptake is low in Fn null fibroblasts compared to control mouse fibroblasts; large amounts of Fn in culture medium enhance bacterial uptake in Fn null fibroblasts but not in control cells. (A) Fn1^flox/flox^ mouse fibroblasts (Fn flox/flox) and Fn1^−/−^ fibroblasts (Fn^−/−^) were infected with S. aureus 6850 or *S. carnosus* TM300(pFNBA4). One hour post infection, extracellular staphylococci were removed by lysostaphin treatment. Subsequently, the host cells were detached and the number of host cells was determined. Host cells were lysed and number of viable intracellular bacteria was assessed by plate counting. Data are means ± SD from three independent experiments. Bacterial uptake of one bacterial strain into the two different fibroblasts cell lines was compared. **, *P < *0.01; ***, *P ≤ *0.001, unpaired *t* test. (B and C) Culture medium of Fn1^flox/flox^ fibroblasts and Fn1^−/−^ fibroblasts was supplemented with Fn at the indicated concentrations. Cells were grown for at least 2 days to reach confluence. Cells were infected for 1 h with either S. aureus 6850 (B) or *S. carnosus* TM300(pFNBA4) (C). Extracellular staphylococci were removed by lysostaphin treatment. Subsequently, the host cells were detached and the number of host cells was determined. Host cells were lysed and the number of viable intracellular bacteria was assessed by plate counting. Numbers of intracellular bacteria in control cells were set to 100%. Data are means ± SD from at least three independent experiments. **, *P ≤ *0.01; ***, *P ≤ *0.001, one-way ANOVA followed by Dunnett’s multiple-comparison test.

To further delineate the role of surface-bound Fn on staphylococcal uptake, mouse fibroblasts incapable of fibronectin production (Fn1^−/−^ fibroblasts) as well as their floxed counterparts Fn1^flox/flox^ expressing Fn were cultured in the absence or presence of exogenously added Fn. In line with the concept of Fn molecules acting as bridging molecules, the absence of Fn in Fn1^−/−^ cells almost abolished uptake of bacteria compared to Fn1^flox/flox^ control cells (Fig. 3A). While in Fn1^−/−^ cells the addition of exogenous Fn dose-dependently enhanced the bacterial uptake efficacy, in Fn1^flox/flox^ cells the uptake was promoted by low doses (10 μg/ml) but inhibited by high doses (50 μg/ml) of exogenous human Fn (Fig. 3B and C). Fluorescence microscopy images revealed the change in Fn association with cells (Fig. S3). This indicates a double-headed relationship between exogenous Fn and bacterial uptake, with basal amounts of surface-displayed Fn being required for staphylococcal uptake and elevated quantities of Fn rather inhibiting staphylococcal ingestion.

10.1128/mBio.01062-21.3 FIG S3 Fn fibril formation by mouse fibroblasts and Fn-null mouse fibroblasts is dependent on the amount of Fn in growth medium. Representative images of immunofluorescence microscopy. Fn1^flox/flox^ control fibroblasts and Fn1^−/−^ fibroblasts were grown in basal medium supplemented with 10% Fn-depleted FBS. Growth medium was supplemented with 0, 10, or 50 μg/ml exogenous Fn during cultivation for 3 days. Cells were stained for Fn (red) and nucleic acid with Hoechst 33342 (blue). Download FIG S3, PDF file, 0.2 MB. Copyright © 2021 Niemann et al. 2021 Niemann et al. https://creativecommons.org/licenses/by/4.0/ This content is distributed under the terms of the Creative Commons Attribution 4.0 International license.

**FIG 4:**
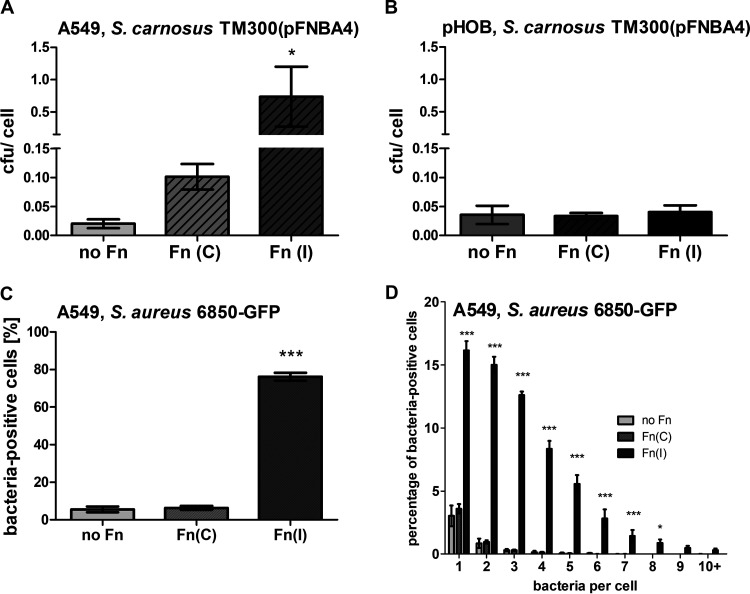
Additional Fn during cultivation of host cells and especially during infection results in a significant increase in bacterial invasion of A549 cells but not of pHOBs. All cells were cultured in growth medium supplemented with Fn-depleted bovine serum. A concentration of 50 μg/ml exogenous Fn was added during cultivation [Fn(C)] (cells were washed before infection) or during infection [Fn(I)]. (A and B) A549 cells (A) or pHOBs (B) were infected with *S. carnosus* TM300(pFNBA4) for 1 h. Afterwards, extracellular staphylococci were removed by lysostaphin treatment. Subsequently, the host cells were detached and the number of cells was determined. Host cells were lysed, and the number of viable intracellular bacteria was assessed by plate counting. Data are means ± SD from three independent experiments. *, *P < *0.05, one-way ANOVA followed by Dunnett’s multiple-comparison test. (C and D) Imaging flow-cytometric analysis (bacterium-positive host cells [C], spot count [D]) of A549, infected with S. aureus 6850-GFP for 1 h, followed by lysostaphin step and fixation. Data represent the means ± SD from three independent experiments. ***, *P ≤ *0.001, one-way ANOVA followed by Dunnett’s multiple-comparison test and two-way ANOVA followed by Bonferroni posttests (spot count).

Having seen the marked influence of exogenously added Fn on bacterial uptake in mouse fibroblasts, the impact of exogenously added Fn on *S. carnosus* TM300(pFNBA4) uptake by A549 and pHOB cells was tested. Fn was supplemented during cell cultivation in one set of experiments as well as during infection in another one. To this end, cells were cultivated in growth medium supplemented with Fn-depleted fetal bovine serum (FBS). Thus, the basal bacterial uptake in the absence of exogenous Fn was not increased in the A549 cells compared to the pHOB cells. However, either addition of Fn to A549 cell culture or particularly during the infection step resulted in a substantial increase of bacterial uptake, whereas these changes were not seen in pHOB cells, regardless of whether the adhesive glycoprotein was added during cultivation or during infection (Fig. 4A and B). Imaging flow-cytometric analysis of S. aureus 6850-GFP showed that addition of Fn during infection resulted in both a highly significant increase in bacterium-positive cells and a highly significant increase in bacteria per cell (Fig. 4C and D).

### Differences in staphylococcal adherence to A549 and pHOB cells.

**FIG 5:**
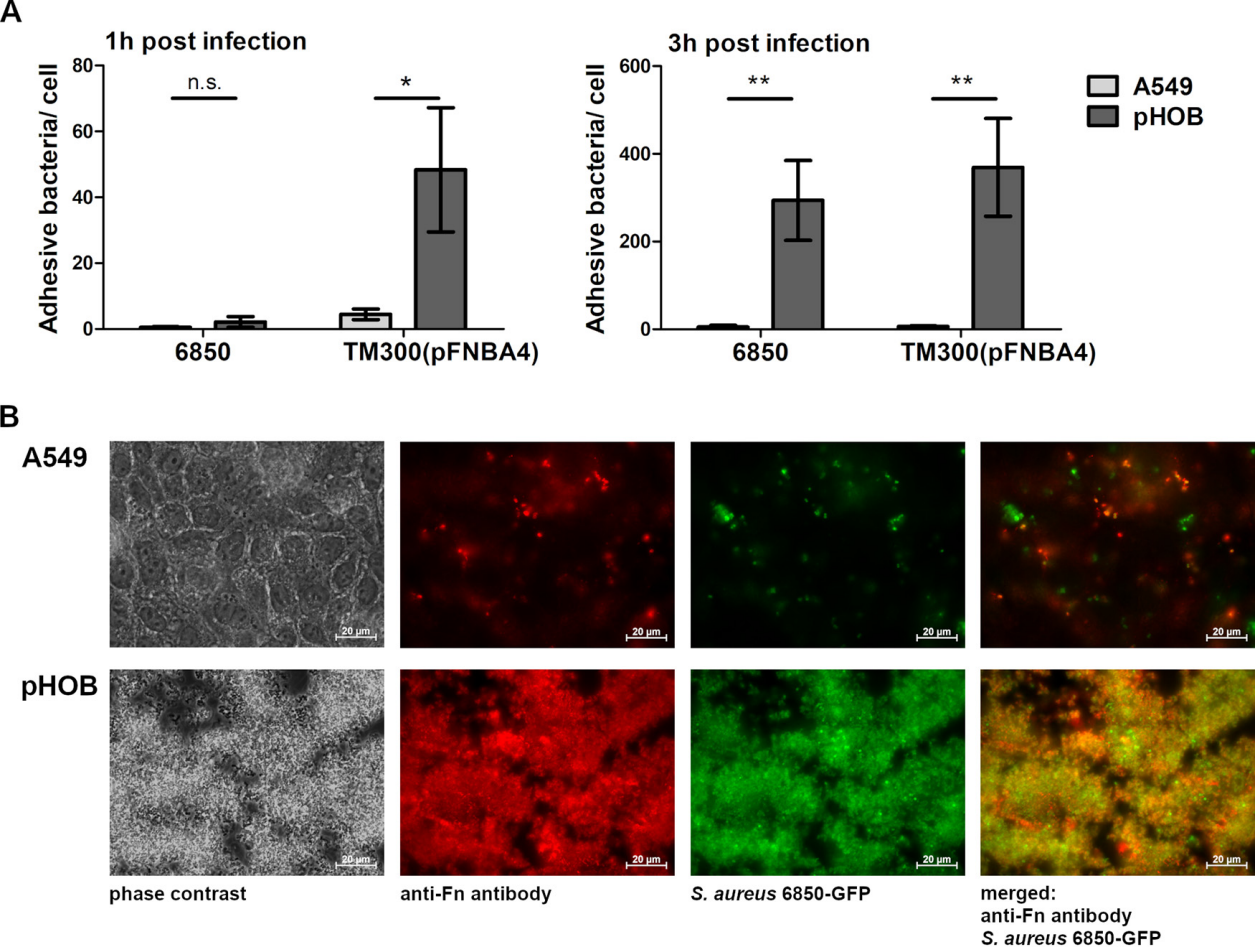
Staphylococci adhere to a large proportion to pHOB. (A) Binding of S. aureus 6850 or *S. carnosus* TM300(pFNBA4) to A549 and pHOB cells after 1 and 3 h of infection (MOI 50). To determine the amount of adhesive bacteria, the quantified amount of bacteria from an experimental setup with lysostaphin (only intracellular bacteria) was subtracted from an experimental setup without lysostaphin (adhesive and intracellular bacteria). The number of host cells was determined, and the number of viable bacteria was assessed by lysing of host cells and plate counting. Data represent the means ± SD from 3 independent experiments. *, *P < *0.05; **, *P ≤ *0.01, unpaired *t* test. (B) Representative fluorescence microscopy images of A549 and pHOB cells infected with S. aureus 6850-GFP (MOI 50) for 3 h, with a washing step but no lysostaphin step; therefore, no distinction between extra- and intracellular bacteria is possible. Images of phase contrast, anti-Fn staining (red), S. aureus 6850-GFP signal (green), and overlay (anti-Fn and S. aureus GFP-signal) are presented.

The differences between bacterial adhesion (Fig. 5) and bacterial uptake to both cell types (Fig. 1A) were analyzed using the lysostaphin protection assay. In essence, to determine the amount of extracellular bacteria (determined as number of CFU/cell) adhering to the eukaryotic cells, the number of bacteria recovered after bacterial incubation with host cells and subsequent lysostaphin treatment (intracellular bacteria) was subtracted from the number of bacteria recovered from the assay without lysostaphin (adherent and intracellular bacteria). Figure 5A depicts the number of S. aureus 6850 or *S. carnosus* TM300(pFNBA4) cells that adhered to either A549 or pHOB cells. As soon as 1 h after incubation, a significantly larger portion of *S. carnosus* TM300(pFNBA4) was found to be bound to the pHOBs compared to the number of bacteria adhering to A549 epithelial cells; after 3 h, such a significant difference was also seen for strain S. aureus 6850. Microscopic examination confirmed these results (Fig. 5B); of note, for the microscopic images, only a thorough washing step with no lysostaphin incubation was performed, i.e., in this assay no distinction between extra- and intracellular bacteria has been made. While no apparent attachment of the bacteria to A549 cells was observed in phase-contrast images, the extracellular bacteria tethered to the pHOB cell layer were clearly visible after 3 h of infection. The merged image of the green fluorescent protein (GFP)-expressing S. aureus 6850 together with the labeled anti-Fn antibody clearly confirmed the association of bacteria with Fn fibrils; bacteria even appear to be surrounded by the adhesive glycoprotein. This impression was also supported by images taken after infection with *S. carnosus* TM300(pFNBA4), which were detected by Hoechst staining (Fig. S4).

10.1128/mBio.01062-21.4 FIG S4 *S. carnosus* TM300(pFNBA4) binds to Fn fibrils of pHOB. Representative images of immunofluorescence microscopy. pHOB were infected with *S. carnosus* TM300(pFNBA4), MOI 50, for 3 h; a thorough washing but no lyostaphin step was performed before fixation. Cells were stained for Fn (red) and nucleic acid (of host cells and bacteria) with Hoechst 33342 (blue). Download FIG S4, PDF file, 0.1 MB. Copyright © 2021 Niemann et al. 2021 Niemann et al. https://creativecommons.org/licenses/by/4.0/ This content is distributed under the terms of the Creative Commons Attribution 4.0 International license.

### Fn fibril formation hampers enhanced bacterial uptake by pHOBs.

As already indicated, large amounts of detergent-insoluble Fn become deposited on pHOBs (compared to A549 epithelial cells) (Fig. 2) but were counterproductive for the uptake process, suggesting that the crude amount of Fn on the cell surface does not necessarily correlate with bacterial uptake efficacy. In fact, large amounts of surface-associated aggregated Fn could interfere with bacterial uptake. To further prove this interpretation in more detail, bacterial uptake assays were performed by following specific manipulations of the Fn matrix surrounding the host cells.

**FIG 6:**
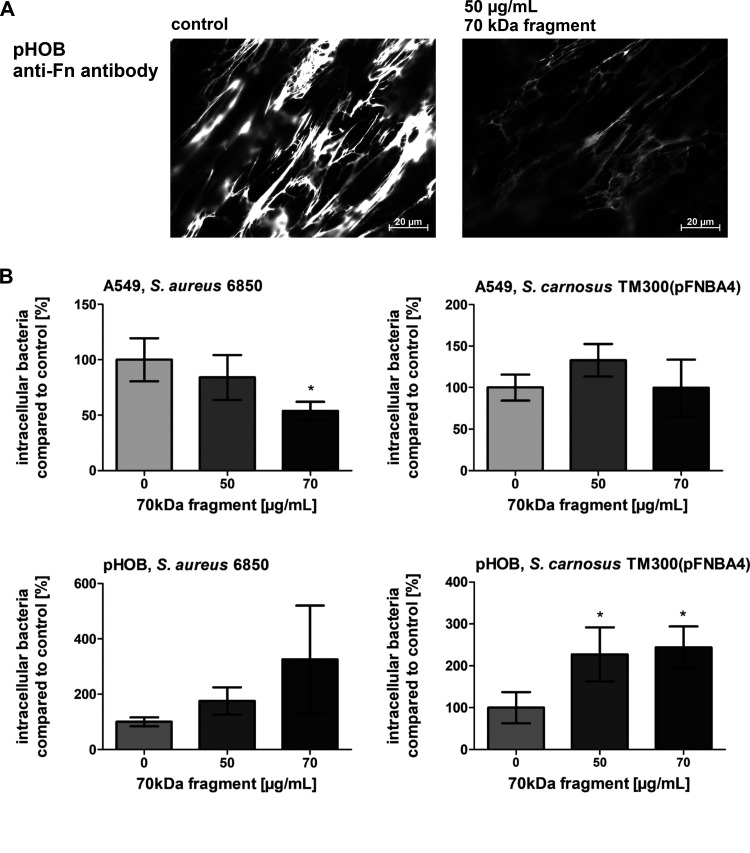
Inhibition of Fn fibril formation increases the bacterial uptake in osteoblasts. Host cells were cultured for 2 days with a 70-kDa fragment of fibronectin to block Fn assembly. (A) Representative immunofluorescence microscopy images of Fn expression on pHOBs detected by anti-Fn antibody. (B) A549 cells and pHOBs cultured with the 70-kDa fragment of Fn as indicated and infected with S. aureus 6850 or *S. carnosus* TM300(pFnBA4) (MOI 50). One hour post infection, extracellular staphylococci were removed by lysostaphin treatment. Subsequently, the host cells were detached and the number of host cells was determined. Host cells were lysed and the number of viable intracellular bacteria was assessed by plate counting. Numbers of intracellular bacteria in non-pretreated cells were set to 100%. Data are means ± SD from 3 independent experiments. *, *P < *0.05, one-way ANOVA followed by Dunnett’s multiple-comparison test.

To inhibit Fn fibril formation, the N-terminal 70-kDa fragment of Fn was employed, which in part blocks the assembly of Fn by occupying the binding sites that are required for Fn self-association (28, 29). Pretreatment of pHOB cells with purified 70-kDa fragment revealed a decline in the formation of thick Fn fibrils as assessed by anti-Fn fluorescence microscopy (Fig. 6A). As is displayed in Fig. 2B, Fn staining of A549 cells was overall rather moderate, and pretreatment with the 70-kDa fragment did not further reduce the low binding signal of the anti-Fn antibody detectable by fluorescence microscopy (data not shown). While pretreatment of A549 cells with the 70-kDa fragment reduced bacterial uptake of S. aureus 6850 [but not of *S. carnosus* TM300(pFNBA4)], it promoted uptake into pHOBs of S. aureus remarkably (>2-fold) and of *S. carnosus* TM300(pFNBA4) significantly (Fig. 6B).

### Influence of silencing Fn expression in A549 and pHOB cells on bacterial uptake.

**FIG 7:**
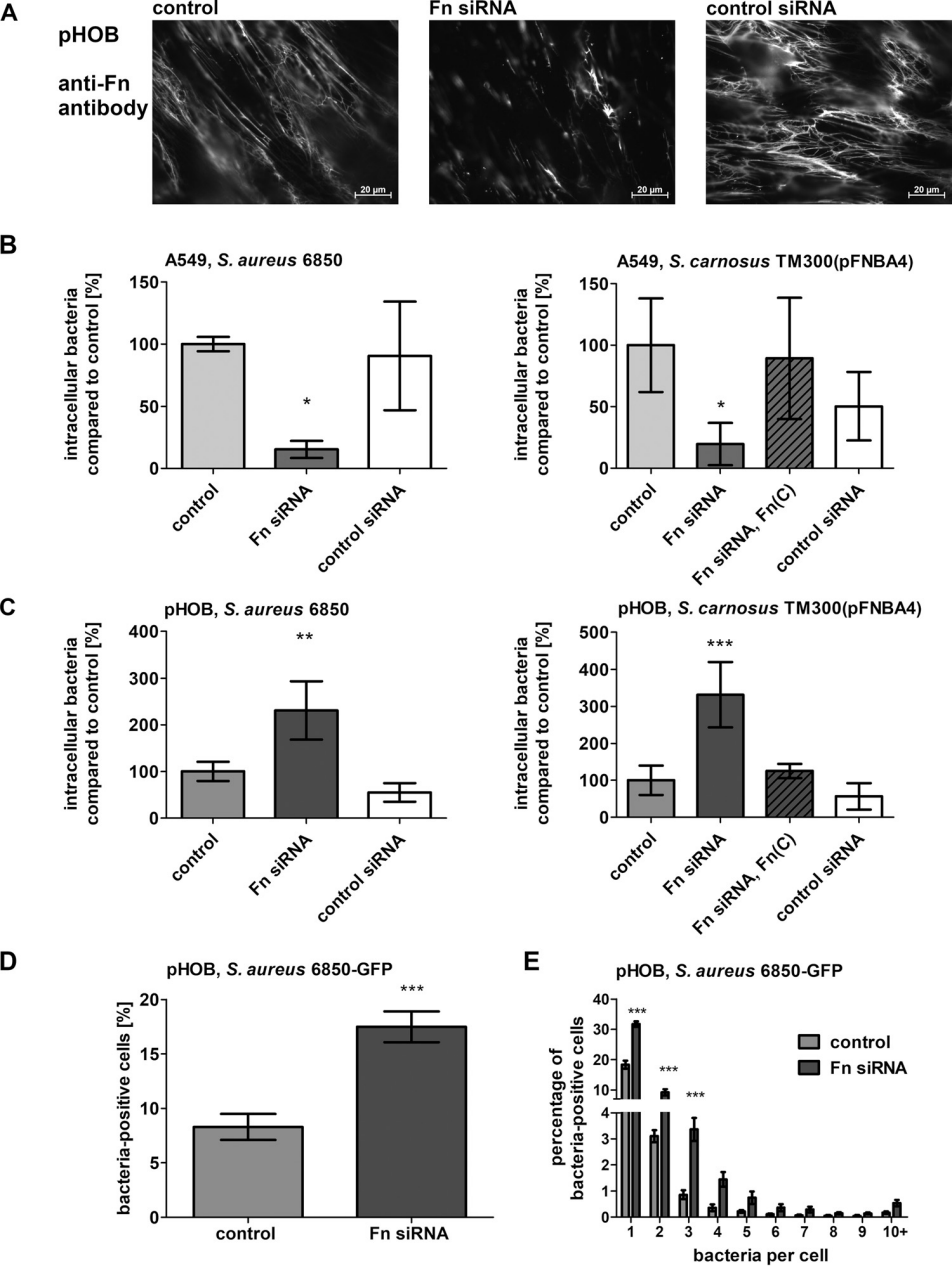
Bacterial uptake in osteoblasts is highly dependent on the amount of Fn covering the cells. A549 and pHOB cells were treated with Fn siRNA or control siRNA for 2 days. (A) Representative immunofluorescence microscopy images of Fn expression on pHOB. Cells were stained for Fn. siRNA treated A549 cells (B) or siRNA treated pHOB (C) were infected with S. aureus or *S. carnosus* TM300(pFNBA4). One hour post infection, extracellular staphylococci were removed by lysostaphin treatment. Subsequently, the host cells were detached and the number of host cells was determined. Host cells were lysed and the number of viable intracellular bacteria was assessed by plate counting. Numbers of intracellular bacteria in control cells were set at 100%. Data are means ± SD from at least three independent experiments. *, *P < *0.05; **, *P ≤ *0.01; ***, *P ≤ *0.001, one-way ANOVA followed by Dunnett’s multiple-comparison test. (D) By imaging flow-cytometric analysis, the uptake of S. aureus 6850-GFP in control pHOB was compared to Fn siRNA-treated cells after 1 h of infection, a lysostaphin step, and fixation. Data represent the means ± SD from three independent experiments. ***, *P ≤ *0.001, unpaired *t* test. (E) By spot counting of imaging flow cytometry data, the amount of bacteria per cell in control pHOB and Fn siRNA-treated cells was compared. Data represent the means ± SD from 3 independent experiments. ***, *P ≤ *0.001, two-way ANOVA followed by Bonferroni posttests.

The expression of Fn was silenced by using specific short interfering RNA (siRNA) to examine the influence of reduced Fn expression in A549 and pHOB cells on their ability to take up bacteria. The efficacy of silencing was proved by Western blotting, confirming a significant reduction of Fn protein, both in the DOC-soluble and in the DOC-insoluble fractions in both cell types (Fig. S5A and B and S6). The pretreatment of cells with a control siRNA did not show any effect. Grown in Fn-free medium, fluorescence microscopic images revealed an altered distribution of extracellular Fn (Fig. 7A). Compared to control cells, either untreated or treated with control siRNA, Fn-silenced cells had remarkably reduced Fn expression but did not completely lose Fn on the cell surface (as already seen by Western blotting). The weak Fn signal on A549 cells was not altered by the corresponding siRNA treatment (not shown).

10.1128/mBio.01062-21.5 FIG S5 Fn siRNA treatment of pHOB and A549 results in a reduction of Fn in the DOC-soluble and DOC-insoluble fractions but has no effect on β1 integrin expression. (A) Representative images of Western blots of the DOC solubility assay. Cells were grown for 3 days and incubated with Fn siRNA or control siRNA for two days (see also Fig. S6). (B) Data represent the means ± SD from Western blot quantification of at least three independent experiments. *, *P < *0.05, one-way ANOVA followed by Dunnett's multiple-comparison test. (C) Fn or control siRNA-treated cells were detached with accutase and incubated with anti-α5 or anti-β1 antibody before flow cytometric analysis. Data represent the means ± SD from three independent experiments. **, *P ≤ *0.01, one-way ANOVA followed by Dunnett's multiple comparison test. Download FIG S5, PDF file, 0.2 MB. Copyright © 2021 Niemann et al. 2021 Niemann et al. https://creativecommons.org/licenses/by/4.0/ This content is distributed under the terms of the Creative Commons Attribution 4.0 International license.

Using the same type of manipulated cells, the bacterial uptake by both cell types (assessed by the lysostaphin protection assay) was studied as a function of silencing of Fn expression. While the uptake of both S. aureus 6850 and *S. carnosus* TM300(pFNBA4) was significantly reduced in A549 cells lacking Fn expression (Fig. 7B), the bacterial internalization efficacy of both strains in pHOB cells was substantially elevated (by 2.5-fold and 3.2-fold, respectively) (Fig. 7C). On pHOB cells treated with siRNA for Fn, imaging flow-cytometric analysis revealed that the increase in number of CFU per cell was an additive effect of more cells carrying bacteria (Fig. 7D) and of a larger number of bacteria, as assessed by spot counting, inside the cells on a per-cell basis (Fig. 7E).

In A549 cells treated with Fn-siRNA, bacterial internalization was restored by exogenous addition of Fn during cell cultivation in the presence of siRNA [Fig. 7B, shown only for *S. carnosus* TM300(pFNBA4)]. In contrast, addition of Fn during cell cultivation/siRNA treatment of pHOB cells significantly reduced the bacterial uptake to a level comparable with the control value [Fig. 7C, shown only for *S. carnosus* TM300(pFNBA4)].

Although the Fn-siRNA treatment could have affected the cellular expression of α5β1-integrins on both cell types, no change in integrin expression was found in differently treated A549 cells. In contrast, siRNA-mediated Fn knockdown resulted in a significant increase in the expression of the α5-subunit in pHOBs (Fig. S5C). However, this increase did not compensate for the overall loss of Fn association with the cell surface (Fig. 7A).

### Relation between silencing of Fn expression and bacterial uptake efficacy on other cells.

To find out whether the observed increase of bacterial uptake by pHOB cells due to a reduction in Fn expression/assembly is a cell-specific phenomenon, other cell types were assessed for this process. To this end, the influence of Fn knockdown was examined in two other cell types with large differences in bacterial uptake efficacy (23): the endothelial cell line EA.hy926, displaying a similar uptake efficacy for S. aureus and A549 cells, and the fibroblast cell line CCD-32Sk, revealing a lower uptake of S. aureus, comparable to the pHOB cells. On EA.hy926 cells, only small quantities of Fn (in both DOC-soluble and DOC-insoluble fractions) were found, comparable to A549 cells, whereas large amounts of Fn were associated with fibroblast CCD-32Sk cells, as already observed for pHOB cells (Fig. S2 and S7A). Immunofluorescence imaging of cells was in accordance with Western blotting for the quantities of cell-associated Fn. The siRNA-mediated Fn knockdown in Ea.hy926 and CCD-32Sk cells resulted in a reduction in immunofluorescently labeled Fn (Fig. S7B) similar to A549 and pHOB cells. In line with previous data obtained with A549 cells, silencing of Fn expression in EA.hy926 cells resulted in a decrease of *S. carnosus* TM300(pFNBA4) uptake, and, similar to the previous data with pHOB cells, in CCD-32Sk cells the siRNA-mediated Fn-knockdown yielded a significant increase in the number of ingested bacteria (Fig. S7C). These findings indicate that bacterial adherence and uptake by host cells appears to depend on the amount and the structure of Fn available, independent of the cell type studied.

10.1128/mBio.01062-21.6 FIG S6 Representative unprocessed Western blots of DOC solubility assay of homogenates of pHOB and A549 after siRNA treatment (see also Fig. S5). Cells were grown for 3 days and incubated with Fn siRNA or control siRNA for two more days. In the lane labeled “soluble Fn,” soluble Fn was applied, which was used as a control. (A) A549 and pHOB, DOC-insoluble fraction. (B) A549 and pHOB, DOC-soluble fraction. Download FIG S6, PDF file, 0.1 MB. Copyright © 2021 Niemann et al. 2021 Niemann et al. https://creativecommons.org/licenses/by/4.0/ This content is distributed under the terms of the Creative Commons Attribution 4.0 International license.

10.1128/mBio.01062-21.7 FIG S7 Augmenting effect on bacterial uptake efficacy by Fn silencing in fibroblast cell line. (A) After 3 days of growth, the DOC-insoluble and the DOC-soluble protein fractions of EA.hy926 endothelial cell line and CCD-32Sk fibroblast cell line were harvested and analyzed by Western blotting (see also Fig. S2). Data are means ± SD from Western blot quantification of three independent experiments. **, *P ≤ *0.01, unpaired *t* test. (B) Representative images of Fn expression on EA.hy926 endothelial cells and CCD-32Sk fibroblasts without and with Fn silencing by Fn siRNA detected by immunofluorescence microscopy using an antibody against Fn (red) and Hoechst 33342 for nucleic acid staining (blue). (C) Lysostaphin protection assay was performed to quantify intracellular bacteria 1 h post infection of either EA.hy926 or CCD-32Sk cells with *S. carnosus* TM300(pFNBA4) without and with Fn silencing by Fn siRNA. Data are means ± SD from three independent experiments. *, *P < *0.05, unpaired *t* test. Download FIG S7, PDF file, 0.3 MB. Copyright © 2021 Niemann et al. 2021 Niemann et al. https://creativecommons.org/licenses/by/4.0/ This content is distributed under the terms of the Creative Commons Attribution 4.0 International license.

### Proteolytic degradation of Fn fibrils increases bacterial uptake in pHOB cells.

**FIG 8:**
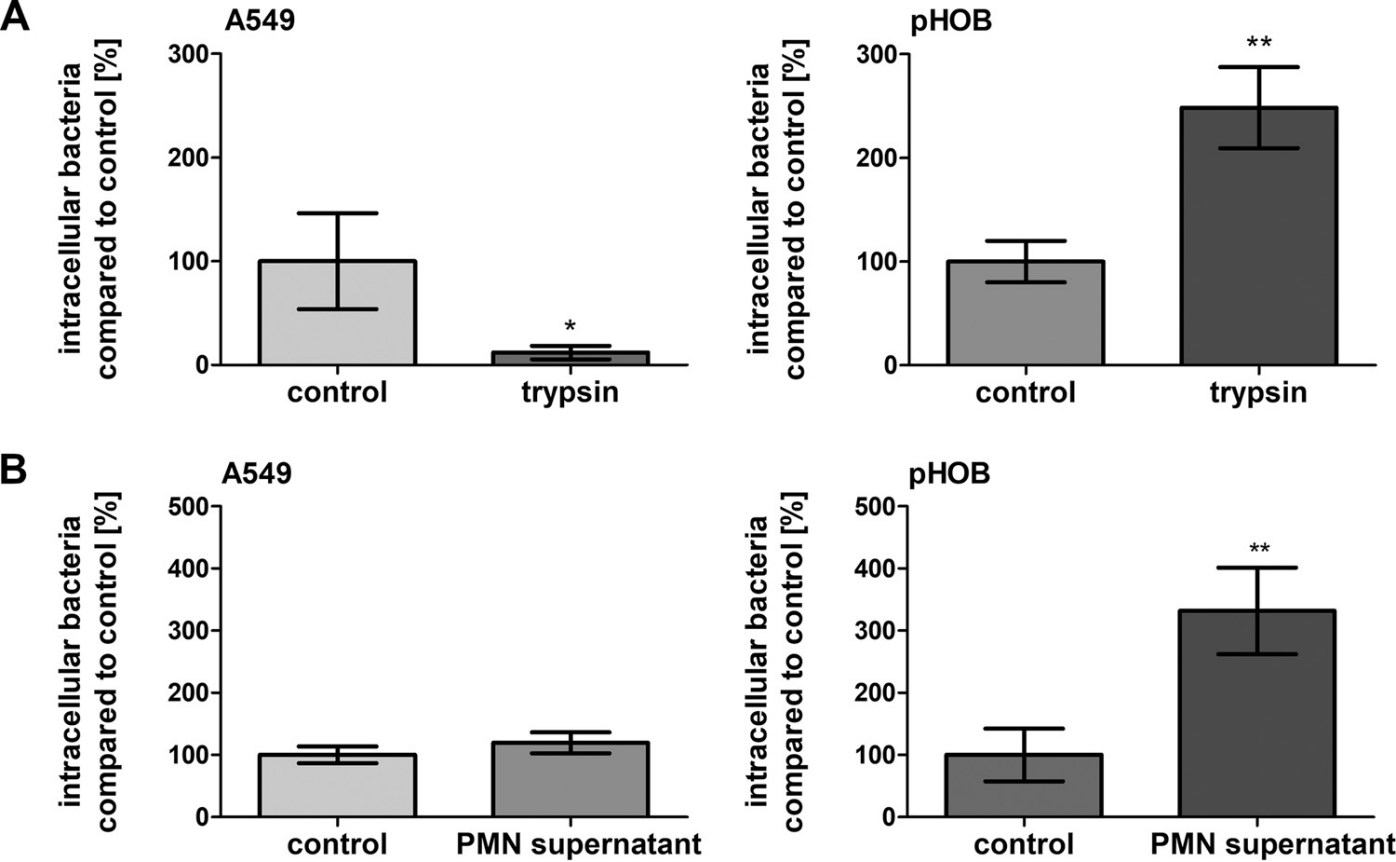
Bacterial uptake by osteoblasts is increased after treatment of cells with trypsin or supernatant of lysed neutrophils. Cells as indicated were treated with either 1 μg/ml trypsin for 30 min (A) or with 10% supernatant of neutrophil lysate (B), destroyed by ultrasound, overnight. Afterwards cells were washed and infected with S. aureus 6850. One hour post infection, extracellular staphylococci were removed by lysostaphin treatment. Subsequently, the host cells were detached and the number of host cells was determined. Host cells were lysed and the number of viable intracellular bacteria was assessed by plate counting. Data are means ± SD from 3 independent experiments. Numbers of intracellular bacteria in control cells were set to 100%; *, *P < *0.05; **, *P ≤ *0.01, unpaired *t* test.

We hypothesized that the formation of supramolecular Fn fibrils modified the bacterial uptake efficacy. Consequently, bacterial ingestion was investigated with pHOB and A549 cells in the absence or presence of trypsin to allow for destroying the Fn fibrillary network. Using microscopic inspection, prior to the bacterial uptake experiments, no visible cell damage following trypsin pretreatment was discernible. However, the dense Fn fibrillary network on pHOB cells was found to be degraded (Fig. S8). The subsequent lysostaphin protection experiments revealed that trypsin-treated pHOB cells contained a significantly higher number of S. aureus 6850 than untreated cells, while trypsin-treated A549 cells took up a smaller number of bacteria (Fig. 8A).

10.1128/mBio.01062-21.8 FIG S8 Fn fibrils are destroyed by trypsin and supernatant of lysed neutrophils. Representative images of phase contrast and immunofluorescence microscopy. pHOB and A549 were grown for two days in normal growth medium supplemented with FBS and then treated either with 1 μg/ml trypsin for 30 min in invasion medium or overnight with 10% supernatant of lysates of polymorphonuclear neutrophils (PMN; destroyed by ultrasound) in basal medium supplemented with 0.21% BSA. Cells were fixed and stained for Fn. Download FIG S8, PDF file, 0.3 MB. Copyright © 2021 Niemann et al. 2021 Niemann et al. https://creativecommons.org/licenses/by/4.0/ This content is distributed under the terms of the Creative Commons Attribution 4.0 International license.

### Role of neutrophil proteases on bacterial uptake in pHOB cells.

Host neutrophils are important effector cells in staphylococcal infections. During infection with S. aureus, a substantial release of neutrophilic proteases (including elastase or cathepsin G) may occur. To test whether such proteases can influence bacterial uptake by A549 and pHOB cells, epithelial and osteoblastic cells were incubated with the supernatant of ultrasound-treated neutrophils overnight. Similar to trypsin, this treatment not only damaged the supramolecular Fn network on A549 and pHOB cells (Fig. S8) but also promoted the uptake of bacteria by the latter cell type, while no influence of neutrophilic proteases was seen on the bacterial internalization capacity of A549 cells (Fig. 8B).

## DISCUSSION

In this study, we demonstrate that the supramolecular structure of Fn molecules deposited on the eukaryotic cell surface plays an essential role in bacterial binding and uptake by these cells. Our findings may not only be of relevance for the uptake mechanism of various staphylococcal strains but also explain the large differences of S. aureus uptake efficacy in different host cell types.

Fn is found in blood plasma, other body fluids, and in association with the extracellular matrix (ECM). It exists in two forms: (i) cellular Fn that is expressed by a variety of cell types and becomes bound to tissue sites where it can assemble into fibrillar matrices and (ii) soluble plasma Fn that is mainly expressed in hepatocytes and secreted into the bloodstream, where it circulates at an appreciable concentration of about 300 μg/ml. A significant portion of tissue Fn originates from plasma as well (30). Fn is secreted as a C-terminally disulfide-linked heterodimer consisting of three structurally defined repeat units (modules). Its supramolecular matrix assembly into an ECM scaffold component proceeds in several steps and is considered a cell-mediated process. Initially, integrin α5β1 interacts with the Arg-Gly-Asp (RGD) sequence in the type III module of Fn with an auxiliary binding site in the neighboring module. In addition, the N-terminal 70-kDa region of Fn interacts with the cell surface. At this stage, integrin-bound Fn is diffusely distributed over the cell surface, and the binding of Fn to multiple integrins results in outside-in signaling, whereby complexes containing α5β1 integrin, focal adhesion kinase (FAK), vinculin, and paxillin begin to assemble, causing integrin clustering as a result of reorganization of the intracellular actin cytoskeleton as well as the extracellular Fn fibrillar assembly.

Initially, these fibrils are organized as short DOC-soluble polymers. The indicated cytoskeletal connections increase cell contractility, which induces conformational changes in Fn as well. Exposed Fn attachment sites and integrin clustering further promote Fn-Fn interactions. Eventually, a stable DOC-insoluble fibrillar matrix is formed (31–33). Five N-terminal type I modules are found to be essential for Fn fibrillogenesis. The N-terminal 70-kDa catheptic fragment comprising these modules binds to cell monolayers with the same affinity as intact Fn and inhibits its assembly but is not incorporated into the detergent-insoluble ECM. The bound 70-kDa fragment colocalizes (incompletely) with preexisting Fn fibrils (28, 34).

In light of these considerations, the staphylococcal uptake by nonprofessional phagocytes such as A549 epithelial and pHOB cells was elucidated in this study with particular respect to the conformational analysis of the external Fn bridging molecule assembly. Obviously, variations between the cell types in the expression of α5β1-integrin as well as of the quantity of cell-bound Fn, hitherto considered of utmost importance for the internalization process of S. aureus (35), were initially assessed. However, a direct correlation between the degree of integrin expression, the quantity of cell-associated Fn, and the bacterial binding and uptake was not compatible with our data utilizing two established host cell types. While pHOBs presented a high expression level of the α5ß1-integrin as well as high levels of cell-associated Fn, their capacity for bacterial uptake was significantly lower than that for A549 cells. Despite a much lower integrin expression level and significantly less cell-associated Fn, these cells exhibited a much higher uptake capacity for bacteria. As deduced from the subsequent experiments in this study, simply the quantity of Fn in association with cells is not relevant for bacterial uptake, but it is the structural type of the adhesive glycoprotein that determines its role as an effective bridging molecule. Thus, the higher the density of the Fn fibrillary meshwork on cells evolves, the less efficient this is for bacterial ingestion.

Fn-floxed fibroblasts also displayed an Fn network on their surface; however, fibrils appeared less thick, and the bacterial uptake in this cell type was high compared to that of pHOB and A549 cells. Proving our hypothesis that Fn is a bridging element necessary for staphylococcal uptake, Fn null fibroblasts displayed very low uptake of bacteria. Also in line with our hypothesis, the external addition of small amounts of Fn to either mouse fibroblast cell line promoted bacterial uptake. However, larger amounts of external Fn further promoted staphylococcal uptake only in Fn null cells, while in the floxed control cells the addition of enhanced amounts of Fn quantities reduced uptake efficiency, a result accompanied by the observation of a microscopically visible denser network of Fn fibrils on the control cells. Thus, it appears that on cell types that form complex fibronectin fibril meshworks on their surfaces, elevated quantities of this bridging molecule rather inhibit staphylococcal ingestion.

Based on these observations, further experiments were performed with A549 cells and pHOB cells. The bacterial uptake of A549 depended on Fn provided in the culture medium, demonstrating that the Fn is necessary to bridge bacteria with the host cells. Moreover, this shows that plasma Fn, which tethers to the host cells and deposits in the extracellular matrix, is fully sufficient for this process (30). Cell-produced cellular Fn is not necessary for bacterial uptake. In contrast, osteoblasts express large amounts of Fn, and even growth in Fn-depleted medium did not reduce fibril formation (Fig. 7). In line with this, further addition of Fn to the culture medium did not lead to an increased uptake of bacteria, whereas the uptake of *S. carnosus* TM300(pFNBA4) by A549 cells was increased upon addition of Fn to the culture medium, compensating for the Fn depletion in the medium. Most strikingly, the addition of Fn to the infection medium resulted in massively increased internalization of *S. carnosus* TM300(pFNBA4) as well, as previously described for S. aureus (11). In contrast, in osteoblasts bacterial uptake was not increased by Fn in the infection medium.

In its soluble form, dimeric Fn represents a compact conformation, whereby the RGD site for interaction with the α5β1-integrin is sterically shielded. It has been shown that binding of S. aureus FnBPA to Fn disrupts specific intermolecular contacts in the N-terminal domain of Fn, resulting in global structural rearrangements at sites distant from the FnBPA binding site on bacteria. Hence, the former cryptic integrin interaction sites become exposed to promote Fn binding to α5β1-integrin (36). This may explain why soluble Fn in the invasion medium so strongly promotes bacterial uptake in A549 cells.

The low uptake of bacteria in osteoblasts is remarkable because the bacteria can fully adhere to the fibronectin fibrils of the cells and are virtually surrounded by fibronectin. The microscopic images of the binding of *S. carnosus* TM300(pFNBA4) to the fibrils confirmed this observation. As a potential artifact by binding of the employed anti-Fn antibody to S. aureus protein A was ruled out, our data demonstrated the intimate interaction of the microorganisms with the eukaryotic cells and their pericellular Fn network despite inefficient uptake.

If excessive Fn fibril formation impedes staphylococcal uptake, a reduction in fibril formation by inhibition of cellular Fn synthesis (in Fn-free culture milieu) could promote bacterial ingestion. In fact, in osteoblasts, silencing of Fn expression led to a reduction in fibril formation and to an increase in bacterial uptake, which could be reversed by exogenous addition of Fn. Experiments with the endothelial cell line EA.hy926 and the fibroblast cell line CCD32-Sk confirmed this assumption, supporting the notion that Fn fibrils largely prevent bacterial uptake in various cell types.

Not only reducing the Fn levels but also the disruption of existing Fn fibrils facilitated bacterial access. Even though fibril formation was only reduced and not completely prevented upon application of the inhibitory 70-kDa fragment, in a concentration-dependent manner it resulted in an elevated bacterial uptake by pHOB cells. Likewise, disruption of the Fn network by trypsin as well as by neutrophilic proteases increased the uptake of bacteria into osteoblasts but not into epithelial cells, which lack such a prominent supramolecular Fn scaffold. During inflammation and infection processes, Fn may be degraded by released proteases, such as matrix metalloproteases or neutrophilic proteases (37, 38). This may result in the fragmentation of Fn fibrils as a “protective envelope” of osteoblasts and fibroblasts allowing S. aureus to enter these cells more easily with the help of endocytosable Fn-fragments. This could, for example, also influence the progression of infections from an acute toward a chronic state.

In summary, S. aureus is taken up in osteoblasts and fibroblasts as well as in epithelial and endothelial cells, presumably by the same main internalization pathway. However, the two groups of cells significantly differ insofar as in osteoblasts and fibroblasts Fn fibrils form a supramolecular scaffold, in which Fn are stably anchored within this matrix. Bacteria find many attachment sites on the Fn fibrils, but they are only poorly taken up. Thus, the supramolecular structure of Fn has a strong impact on host cell uptake efficacy. At this point, we can only speculate about the reasons for this effect. One reason could be that S. aureus FnBPs promote adhesion of S. aureus to soluble Fn via strong molecular bonds due to the tandem β zippers formed between FnBPs and Fn. Much weaker bonds were observed with fibrillar Fn, presumably due to an increase in the distance between the bacterial binding sites of Fn by stretching of module I during fibril assembly (39, 40). This could hinder the uptake process. Alternatively, bacterial uptake and fibril formation use the same “cellular force-generating machinery” (41). Both processes start with Fn binding to α5ß1-integrin and outside-in signaling, followed by integrin clustering due to cytoskeletal rearrangement. Thus, the bacterial uptake process is competed for by Fn fibril formation on osteoblasts. Furthermore, the uptake is hampered due to the fact that the internalization of bacteria also requires the uptake of the Fn-integrin complexes (15). This could be achieved by disrupting these complexes, e.g., by proteolytic degradation. The proposed mechanisms may contribute to the observed effect in a concerted action. The established term that Fn is both “necessary and sufficient” for staphylococcal invasion of eukaryotic cells (11) thus needs to be modified into a more precise formulation that the Fn as such is necessary for bacterial binding to host cells, but only the supramolecular structure of Fn determines uptake.

As a potential experimental bias it may be reasoned that A594 cells (as well as the EA.hy926 cells, a fusion line of A549 cells and primary human vein endothelial cells [HUVECs]) could behave differently than the (primary) osteoblasts and the (nontumorous) fibroblasts. In fact, tumor-derived cell lines may show less Fn expression or Fn fibrils than primary cells (42, 43). However, already previously (23) we have reported that HUVECs are capable of a similar amount of uptake of S. aureus compared to A549 cells. In the current study, moreover, A549 cells and primary HUVECs were compared with respect to bacterial uptake: results revealed that the tumorous nature of the A549 cells (or the EA.hy926 cells) does not explain the difference in bacterial uptake when comparing the endothelial/epithelial cell lines with the fibroblasts/osteoblasts used in this study (also supported by fluorescence microscopy observations on Fn decoration of the respective cells) (data available at https://doi.org/10.6084/m9.figshare.14938056).

Our observations do not allow yet for a clear extrapolation of the data toward the *in vivo* situation. Plasma Fn levels are subject to wide fluctuations, with reduced plasma Fn levels associated with acute inflammation, surgical trauma, and disseminated intravascular coagulation (44). The distribution of cellular integrins *in vitro* (two-dimensional [2D]) differ from the 3D *in vivo* situation, and integrins are also subject to constant change *in vivo*. For example, they are exposed in osteoblasts, particularly after fractures, to allow cell contact and wound closure (45). Recently, it has been described that Fn also has an intrinsic capacity for self-assembly into amyloid-like structures under certain conditions, and amyloid-like Fn deposits also have been found in the liver of patients (46–48). It has not yet been investigated whether S. aureus interacts differently with these Fn aggregates than with soluble or fibrillar Fn. Moreover, other glycoproteins of the ECM, such as various collagens, which were not investigated here but which interact with Fn fibrils (49), could modify bacterial binding to and uptake by host cells.

The demonstrated role of the supramolecular structure of ECM components for the uptake of S. aureus into host cells might explain *in vivo* differences between courses of bacterial infections and the localization of bacteria in different clinical settings. For instance, early invasion of endothelial cells is a prerequisite for the initiation of acute staphylococcal endocarditis (50, 51), while the invasion of osteoblasts by staphylococci might be important for the chronic course of bacterial osteomyelitis (4, 6, 10, 52). In the light of such considerations, our findings may not only be relevant for our understanding of the involved pathogenetic mechanisms but also may have important implications for the prevention and treatment of invasive staphylococcal diseases.

## MATERIALS AND METHODS

### Ethical statements.

The isolation of human primary osteoblasts and human blood collection of healthy volunteers was approved by the local ethics committee (Az. 2012-032-f-S; Ethik-Kommission der Ärztekammer Westfalen-Lippe und der Medizinischen Wilhelms-Universität Münster). Written informed consent was obtained.

### Bacterial strains and culture.

The strains S. aureus 6850 (24, 25), the green fluorescent protein (GFP)-expressing S. aureus 6850 (53), and *S. carnosus* TM300(pFNBA4), an FnBPA-expressing *S. carnosus* TM300 strain (12), were used. Bacteria from overnight culture in tryptic soy broth (TSB) (shaking conditions, 37°C) were adjusted to an optical density at 578 nm (OD_578_) of 0.1 in TSB. After 3 h of growth under the same conditions, the bacteria were adjusted to an OD_578_ of 1 and stored in aliquots at −20°C until use. From a previously frozen aliquot, the number of CFU was determined after serial dilutions on blood agar plates of the bacterial suspensions. In case of *S. carnosus* TM300(pFNBA4), TSB supplemented with 10 μg/ml chloramphenicol was used.

### Cell culture.

A549 cells (ACC 107; DSMZ GmbH, Germany), a human alveolar epithelial cell line, and the fibroblast cell line CCD-32SK (ATCC CRL-1489; LGC, Germany), were cultured in Dulbecco's modified Eagle medium (DMEM; Sigma-Aldrich) supplemented with 10% fetal bovine serum (FBS superior; S0615; Sigma-Aldrich). The human endothelial cell line EA.hy926 (ATCC CRL-2922; LGC, Germany) was cultured in DMEM supplemented with 10% FBS and 1× HAT (Gibco/ThermoFisher). A mouse Fn1-ko fibroblast cell line (Fn1^−/−^ cells) and the *Fn1^flox/flox^* control cells (54) were a kind gift from Reinhard Fässler, MPI of Biochemistry, Martinsried, Germany. The cells were cultured in DMEM (Gibco/ThermoFisher) supplemented with 10% FBS. Primary human osteoblasts were generated from normal trabecular bone specimens as described previously (55), with some modifications. Briefly, the bone material was isolated using a sharp spoon. The resulting bone chips were washed with phosphate-buffered saline (PBS) and treated with trypsin-EDTA. Subsequently, the chips were seeded in MEM-alpha modification (M8042; Sigma-Aldrich) supplemented with 2 mM l-glutamine, 0.2 mM l-ascorbic acid 2-phosphate, 10 mM β-glycerophosphate disodium salt hydrate, and 10 nM dexamethasone (all from Sigma-Aldrich). All cells were cultured at 37°C and 5% CO_2_.

The cells were seeded in 12-well plates 2 or 3 days before the experiments and were used at 90 to 100% confluence. On the day of the experiment, cells from one spare well of the plate were detached with trypsin-EDTA and the cell number was determined using an automated cell counter (TC20; Bio-Rad).

### Invasion assay-lysostaphin protection assay.

Host cells were infected with a multiplicity of infection (MOI) of 50 in invasion medium (basal medium depending on the cell type, 1% human albumin (Kedrion), and 10 mM HEPES and incubated for 1 or 3 h (as indicated) at 37°C and 5% CO_2_. After washing, extracellular bacteria were eliminated by lysostaphin (20 mg/ml; WAK-Chemie) for 30 min. Cells were detached by trypsin-EDTA. The number of cells was determined using an automated cell counter (TC20; Bio-Rad). Subsequently, the cells were centrifuged and the pellet was dissolved in ice-cold water to allow cell lysis. To determine the number of CFU, serial dilutions of the cell lysates were cultured overnight on Columbia blood agar at 37°C. Invasion was expressed as the mean number of CFU per host cell.

### Manipulation of cellular invasion.

Several bacterial uptake assays were performed in which the Fn matrix surrounding the cells was previously manipulated.

**(i) Fibronectin.** For some experiments, as indicated, the fibronectin present in the FBS in the culture medium was depleted by affinity chromatography. For this, a column filled with 5 ml gelatin Sepharose 4B (GE Healthcare/ThermoFisher) was used. A volume of 10 ml of FBS superior was passed over the column four times. The efficiency of the depletion was validated by Western blotting. For other experiments, exogenous human plasma Fn (Roche/Sigma-Aldrich) was added (concentration as indicated) to the culture medium when seeding the cells for experiments or to the invasion medium.

**(ii) siRNA knockdown of Fn.** Fn small interfering RNA (siRNA) and negative-control siRNA were purchased from Dharmacon/Horizon Discovery, Cambridge, United Kingdom (SMARTPool ON-TARGETplus human FN1, L-009853-00-0005, and ON-TARGETplus non-targeting siRNA, D-001810-10-05). Cells were seeded in 12-well plates in culture medium containing Fn-depleted FBS the day before transfection. Cells were approximately 60% confluent at transfection. Cells were transfected with siRNA (10 nM) using Lipofectamine RNAiMAX reagent (ThermoFisher) according to the manufacturer's instructions; RNAiMAX reagent and siRNA were diluted in Opti-MEM (31985047; Gibco/ThermoFisher) before being added to the cells. Cells were incubated at 37°C and 5% CO_2_ for 2 days before starting the experiment. Efficiency of knockdown was confirmed by Western blot analysis. In the case of addition of Fn during growth with siRNA transfection, glycoproteins were added to the culture medium 4 to 5 h after addition of siRNA.

**(iii) Fragment of 70 kDa.** To inhibit Fn fibril formation, a 70-kDa amino-terminal fragment of Fn (F0287; Sigma-Aldrich) was added immediately after seeding of cells. Cells were grown for 2 days, and the growth medium containing the 70-kDa fragment was replaced once.

**(iv) Trypsin.** Thirty minutes prior to infection, 1 μg/ml trypsin-TPCK (T1426; Sigma-Aldrich) in infection medium was added to the cells. Afterwards, cells were washed once with PBS and were infected with the bacteria in fresh invasion medium.

**(v) Supernatant of lysed neutrophils.** Human polymorphonuclear cells (neutrophils) were freshly isolated from Na citrate‐treated blood of healthy donors and isolated by dextran sedimentation and Ficoll‐Paque density gradient. Neutrophils were resuspended at a final density of 1 × 10^6^ cells/ml in basal medium (matching the host cell type to be treated with the neutrophil supernatant), treated with ultrasound to lyse the cells, and pelleted by centrifugation. Finally, 10% supernatant was added to the host cells in basal medium supplemented with 0.21% bovine serum albumin (BSA) (56). After 18 h of incubation, the medium was removed and replaced by invasion medium, and cells were infected with bacteria.

### Western blot analysis.

To assess the amount of fibronectin on host cells and to analyze matrix assembly, we followed the protocol of Wierzbicka‐Patynowski et al. (57) using a DOC solubility assay. The assay is based on the insolubility of stable Fn matrix in 2% DOC detergent. Cells were lysed in DOC lysis buffer (1% DOC, 20 mM Tris-HCl, pH 8.5, 2 mM N-ethylmaleimide, 2 mM iodoacetic acid, 2 mM EDTA, and 2 mM phenylmethylsulfonyl fluoride [PMSF]), passed through a 26-gauge needle, and centrifuged at 18,400 × *g* for 20 min at 4°C. DOC-soluble material (supernatant) was retained, and the DOC-insoluble pellet was dissolved in SDS-solubilization buffer (1% [wt/vol] SDS, 20 mM Tris-Cl [pH 8.8], 2 mM EDTA, 2 mM iodoacetic acid, 2mM N-ethylmaleimide, and 2 mM PMSF). Protein concentration of DOC-soluble fraction was determined by bicinchoninic acid protein assay kit (ThermoFisher). Protein concentration of DOC-insoluble samples was based on protein concentration in the corresponding DOC-soluble fraction. Both fractions were separated by SDS-PAGE (4 to 15% precast polyacrylamide gel; Bio-Rad) under reduced conditions and analyzed by Western blotting (anti fibronectin antibody; rabbit polyclonal; ab2413; Abcam), anti-tubulin antibody (mouse monoclonal; T6199; Sigma-Aldrich), PageRuler plus prestained protein ladder, 10 to 250 kDa (26619; ThermoFisher). Images of Western blots were obtained with a ChemiDoc touch imaging system, and the quantification was performed with Image Lab 6.1 software (both Bio-Rad).

### Flow-cytometric determination of α5β1 integrin expression.

Confluent cells were detached by accutase (Sigma-Aldrich), taken up in PBS supplemented with 10% FBS, and pelleted at 500 × *g*, 5 min, room temperature (RT). Cell pellets were resuspended in invasion medium and stained with phycoerythrin (PE)-labeled anti-integrin α5 monoclonal antibody (12-4900-42; eBioscience/ThermoFisher) or PE-labeled anti-integrin ß1 monoclonal antibody (555443; BD Pharmingen) for 30 min at RT in the dark. Cells were washed, and antibody binding to integrin was analyzed by flow cytometry (Accuri C6; Becton, Dickinson). Cells were gated by forward scatter/side scatter characteristics, and data were obtained as medians from the fluorescence channel. A total of 5,000 gated events were analyzed for each data point. Data was processed by the C6 Analysis Software.

### Microscopic analysis.

For microscopic analysis, cells were seeded on imaging dishes with cover glass bottom (MoBiTec). Cells were treated as indicated. Afterwards, the cells were fixed in 4% formaldehyde (Histofix; Carl Roth) for 20 min at RT. Unspecific binding was blocked by PBS supplemented with 1% BSA for 30 min at RT prior to staining for 30 min at RT with monoclonal anti-Fn antibody (MA5-11981; ThermoFisher; in PBS with 0.1% BSA). Subsequently, cells were washed and stained with Alexa Fluor-568-conjugated anti-mouse secondary antibody (A11004; ThermoFisher; 30 min at RT in the dark; in PBS with 0.1% BSA). Simultaneously, host cells (and bacteria) were counterstained with Hoechst 33342 (H3570; ThermoFisher). Mouse IgG antibody (555746; PD Pharmingen) served as a control.

Cells were analyzed by fluorescence microscopy (Axio Observer.Z1; Carl Zeiss; equipped with filter set 38 HE [EX BP 470/40, BS FT 495, EM BP 525/50], filter set 43 HE [EX BP 550/25, BS FT 570, EM BP 605/70], and filter set 49 [EX G 365, BS FT 395, EM BP 445/50] and 100×/numeric aperture 1.3 Plan-Neofluar objective). Pictures were documented with an AxioCam MRm camera and processed using Zeiss AxioVision or ZEN 3.2 (blue edition) software.

### Imaging flow-cytometric analysis.

Internalization of bacteria in host cells was assessed using an ImageStream Mk II (Amnis) imaging flow cytometer equipped with an EDF (enhanced depth of field) module and IDEAS analysis software (Amnis), allowing flow-cytometric acquisition and microscopic visualization of target cells. As indicated, cells were infected with S. aureus 6850-GFP for 1 h, followed by lysostaphin treatment. Thereafter, cells were detached using trypsin-EDTA and fixed with 4% formaldehyde before data acquisition. Analysis included gating on single host cells and subsequent determination of internalized GFP-expressing bacteria. Additionally, spot-counting analysis was performed to obtain data on amount of S. aureus 6850-GFP on a per-cell basis.

### Statistical analysis.

The data are expressed as means ± standard deviations (SD). Statistical analyses were performed with Prism (GraphPad Software) using two-tailed unpaired *t* test or one-way analysis of variance (ANOVA), followed by Bonferroni or Dunnett’s multiple-comparison procedure versus the control as appropriate. Spot count data obtained by imaging flow-cytometric analysis was analyzed using two-way ANOVA followed by Bonferroni posttest. A *P* value of <0.05 was accepted as significant.

### Data availability.

The data that support the findings of this study are openly available at figshare at https://doi.org/10.6084/m9.figshare.14938056.
