# Correction for Niemann et al., “More Is Not Always Better—the Double-Headed Role of Fibronectin in *Staphylococcus aureus* Host Cell Invasion”

**DOI:** 10.1128/mbio.02921-23

**Published:** 2023-11-30

**Authors:** Silke Niemann, Minh-Thu Nguyen, Johannes A. Eble, Achmet I. Chasan, Maria Mrakovcic, Klaus T. Preissner, Steffen Roßlenbroich, Georg Peters, Mathias Herrmann

## AUTHOR CORRECTION

Volume 12, no. 5, e01062-21, 2021, https://doi.org/10.1128/mbio.01062-21. Ralph T. Böttcher appeared as a coauthor but should be removed. The byline should appear as given in this correction.

