# Supplementary material for: More Is Not Always Better—the Double-Headed Role of Fibronectin in Staphylococcus aureus Host Cell Invasion
Source: mBio. 2021 Oct 19;12(5):e01062-21. doi: 10.1128/mBio.01062-21 (PMC8524341; doi:10.1128/mBio.01062-21)
Supplement: FIG S1 [file mbio.01062-21-sf001.pdf]

Fig. S1

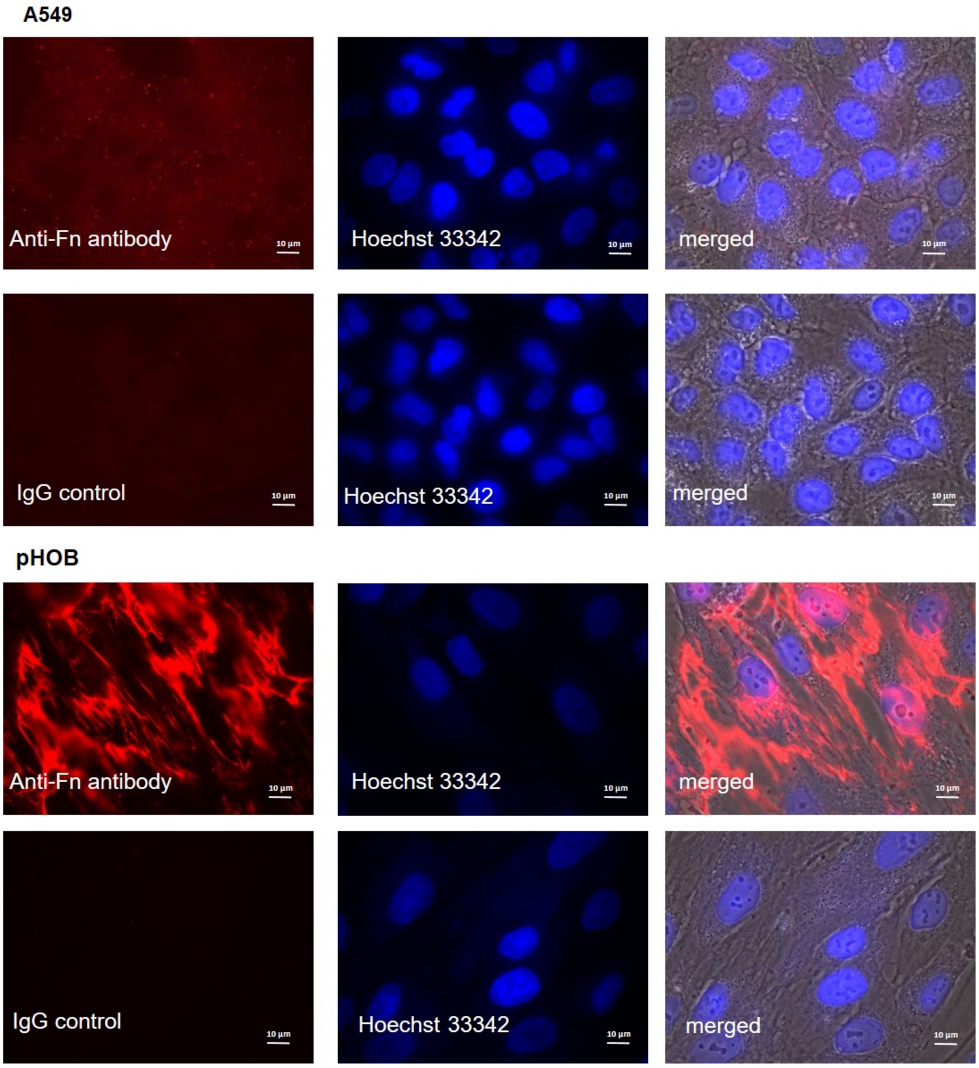

Fig. S1: Primary osteoblast express more Fn on the cell surface as compared to A549 cells. Representative images of Fn expression on A549 and pHOB cells visualized by immunofluorescence microscopy using an antibody against Fn or IgG control antibody (red) and Hoechst 33342 for nucleic acid staining (blue).
