# Supplementary material for: More Is Not Always Better—the Double-Headed Role of Fibronectin in Staphylococcus aureus Host Cell Invasion
Source: mBio. 2021 Oct 19;12(5):e01062-21. doi: 10.1128/mBio.01062-21 (PMC8524341; doi:10.1128/mBio.01062-21)
Supplement: FIG S2 [file mbio.01062-21-sf002.pdf]

Fig. S2

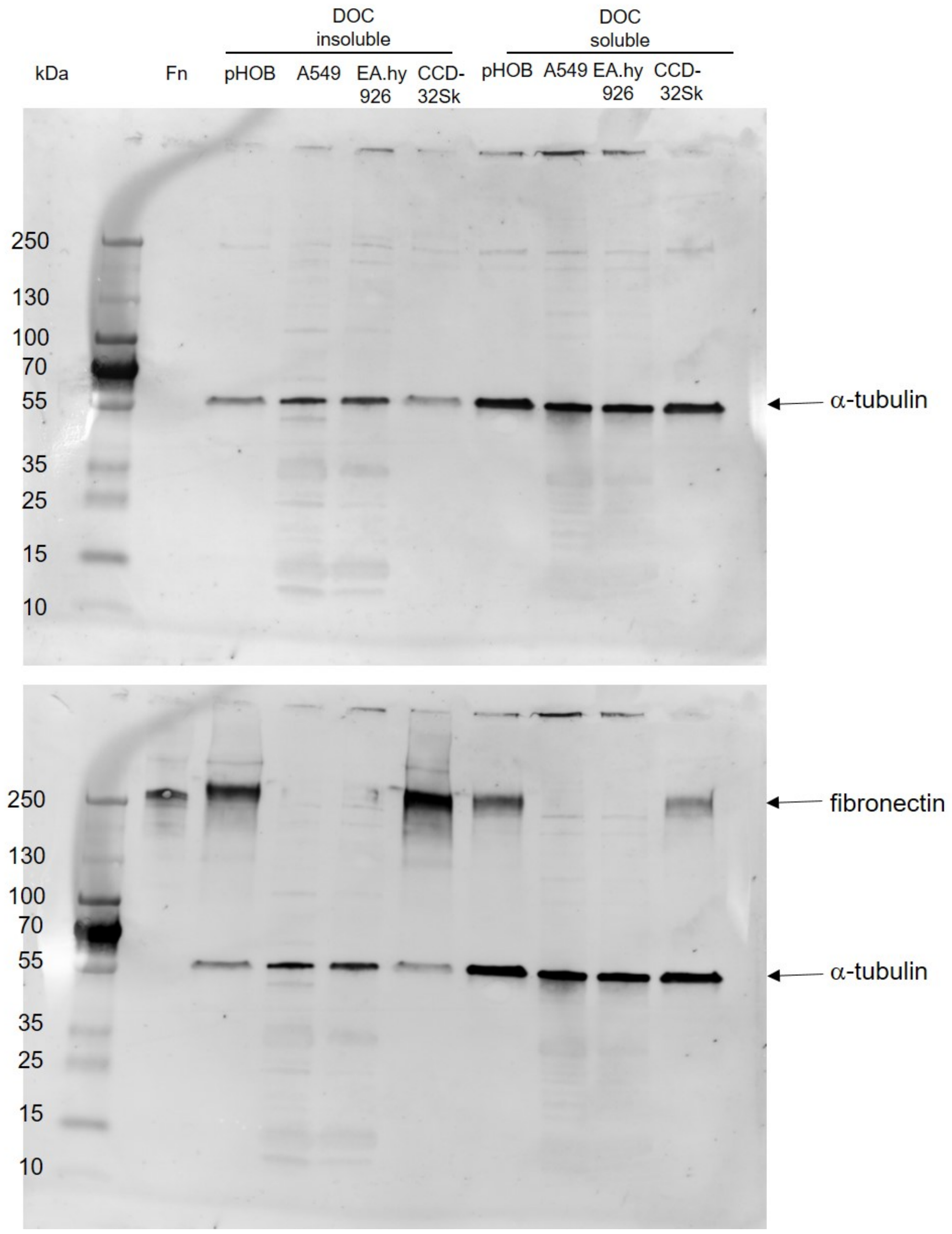

Fig. S2: Representative unprocessed Western blot of DOC solubility assay of homogenates of pHOB, A549, EA.hy926 and CCD-32Sk. DOC-insoluble and DOC-soluble fraction of cell homogenates were separated by SDS-PAGE under reduced conditions and analyzed by Western blotting. In the lane labeled "Fn", soluble Fn was applied which was used as control. The same blot is shown twice. The upper image shows the bands after staining with anti-α-tubulin antibody (loading control). Slight nonspecific bands almost at the position of the fibronectin can be detected. The lower panel additionally shows the Fn bands after anti-Fn antibody staining. Original Western blot on which Fig. 2C and Fig. S7A are based.
