# Supplementary material for: More Is Not Always Better—the Double-Headed Role of Fibronectin in Staphylococcus aureus Host Cell Invasion
Source: mBio. 2021 Oct 19;12(5):e01062-21. doi: 10.1128/mBio.01062-21 (PMC8524341; doi:10.1128/mBio.01062-21)
Supplement: FIG S3 [file mbio.01062-21-sf003.pdf]

Fig. S3

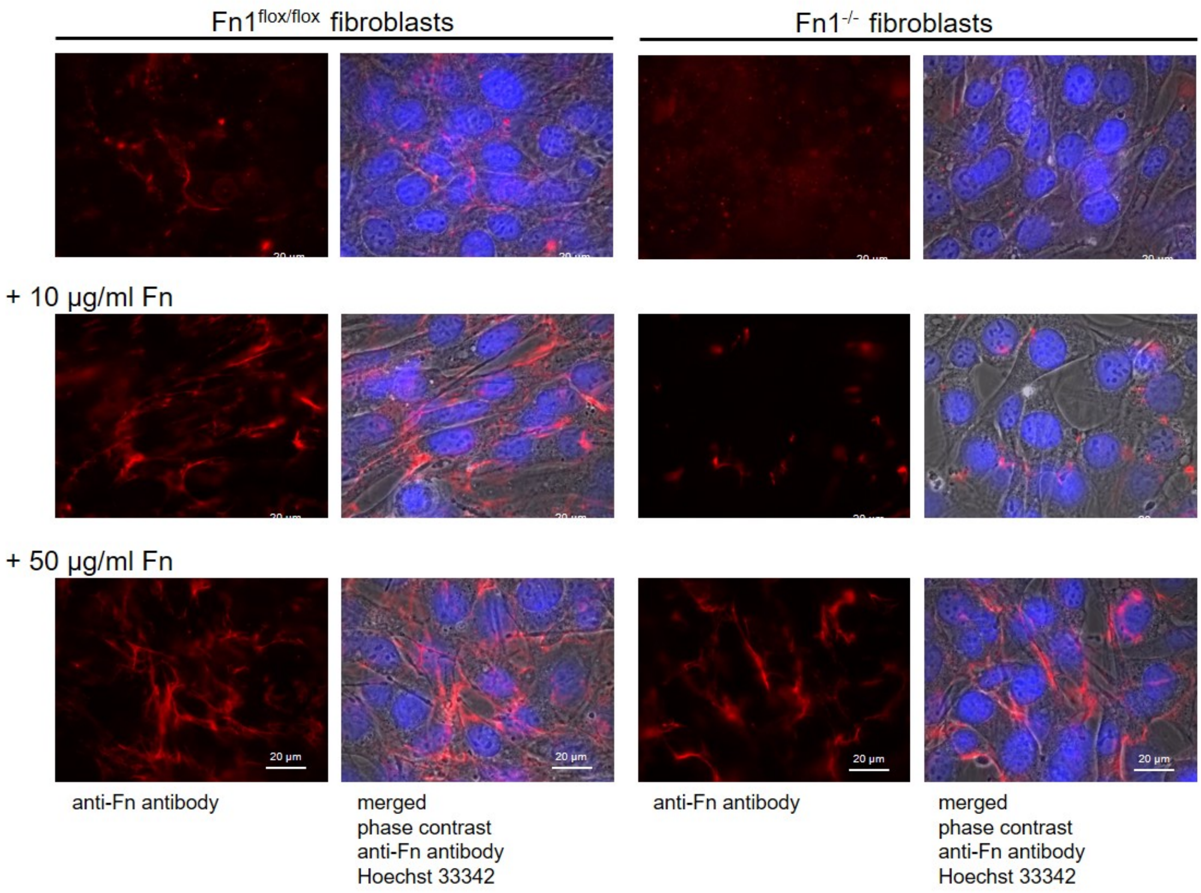

Fig. S3: Fn fibril formation by mouse fibroblasts and Fn-null mouse fibroblasts is dependend on the amount of Fn in growth medium. Representative images of immunofluorescence microscopy. $Fn1^{flox/flox}$ control fibroblasts and $Fn1^{-/-}$ fibroblasts were grown in basal medium supplemented with 10% Fn-depleted FBS. Growth medium was supplemented with 0, 10 or 50 µg/ml exogenous Fn during cultivation for three days. Cells were stained for Fn (red) and nucleic acid with Hoechst 33342 (blue).
