# Supplementary material for: More Is Not Always Better—the Double-Headed Role of Fibronectin in Staphylococcus aureus Host Cell Invasion
Source: mBio. 2021 Oct 19;12(5):e01062-21. doi: 10.1128/mBio.01062-21 (PMC8524341; doi:10.1128/mBio.01062-21)
Supplement: FIG S4 [file mbio.01062-21-sf004.pdf]

Fig. S4

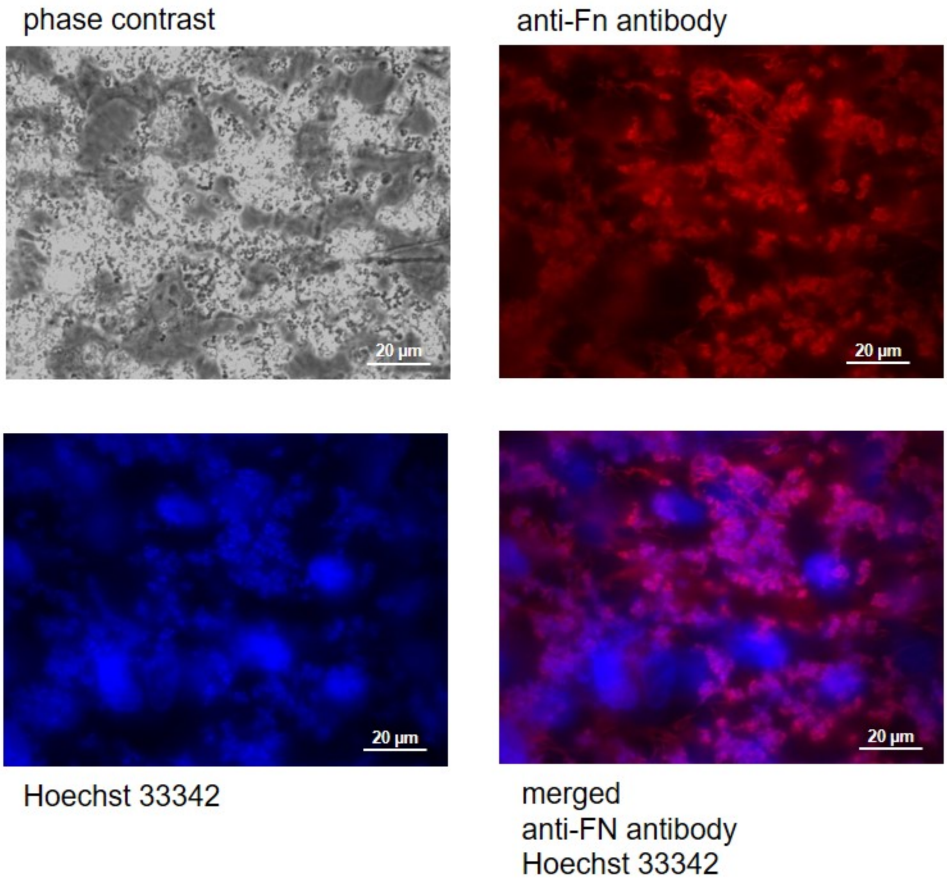

Fig. S4: *S. carnosus* TM300(pFNBA4) binds to Fn fibrils of pHOB. Representative images of immunofluorescence microscopy. pHOB were infected with *S. carnosus* TM300(pFNBA4), MOI50, for three hours; a thorough washing but no lyostaphin step was performed before fixation. Cells were stained for Fn (red) and nucleic acid (of host cells and bacteria) with Hoechst 33342 (blue).
