# Supplementary material for: More Is Not Always Better—the Double-Headed Role of Fibronectin in Staphylococcus aureus Host Cell Invasion
Source: mBio. 2021 Oct 19;12(5):e01062-21. doi: 10.1128/mBio.01062-21 (PMC8524341; doi:10.1128/mBio.01062-21)
Supplement: FIG S5 [file mbio.01062-21-sf005.pdf]

Fig. S5

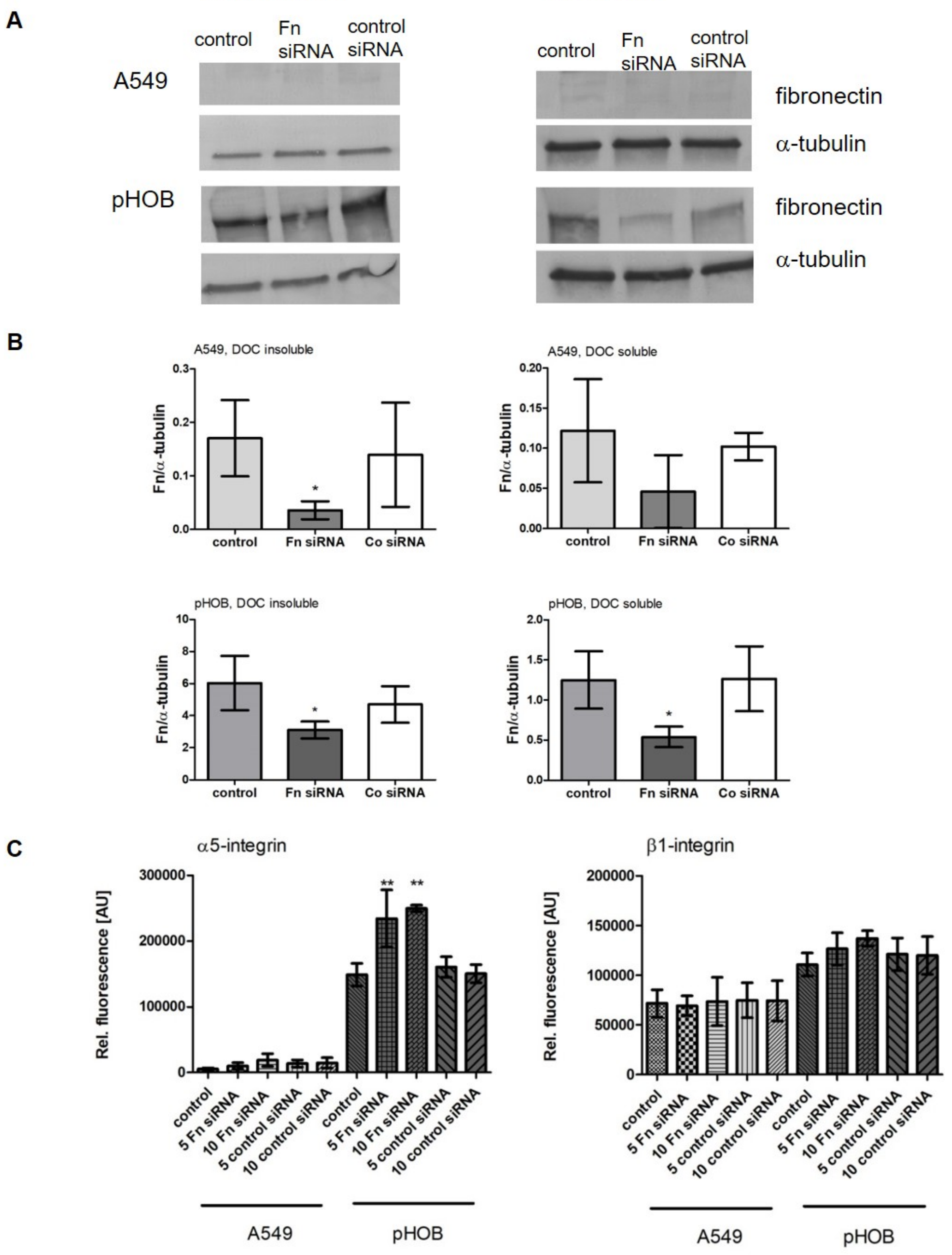

Fig. S5: Fn siRNA treatment of pHOB and A549 results in a reduction of Fn in the DOC-soluble and DOC-insoluble fractions, but has no effect on β1 integrin expression. (A) Representative images of Western blots of the DOC solubility assay. Cells were grown for three days and incubated with Fn siRNA or control siRNA for two days (see also Fig. S6). **(B)** Data represent the mean ± SD of Western blot quantification of at least three independent experiments. * $p<0.05$, one-way ANOVA followed by Dunnett's multiple comparison test. **(C)** Fn or control siRNA treated cells were detached with accutase and incubated with anti-α5 or anti-β1 antibody before flow cytometric analysis. Data represent the mean ± SD of three independent experiments. ** $p\leq0.01$, one-way ANOVA followed by Dunnett's multiple comparison test.
