# Supplementary material for: More Is Not Always Better—the Double-Headed Role of Fibronectin in Staphylococcus aureus Host Cell Invasion
Source: mBio. 2021 Oct 19;12(5):e01062-21. doi: 10.1128/mBio.01062-21 (PMC8524341; doi:10.1128/mBio.01062-21)
Supplement: FIG S6 [file mbio.01062-21-sf006.pdf]

## Fig. S6

**A**

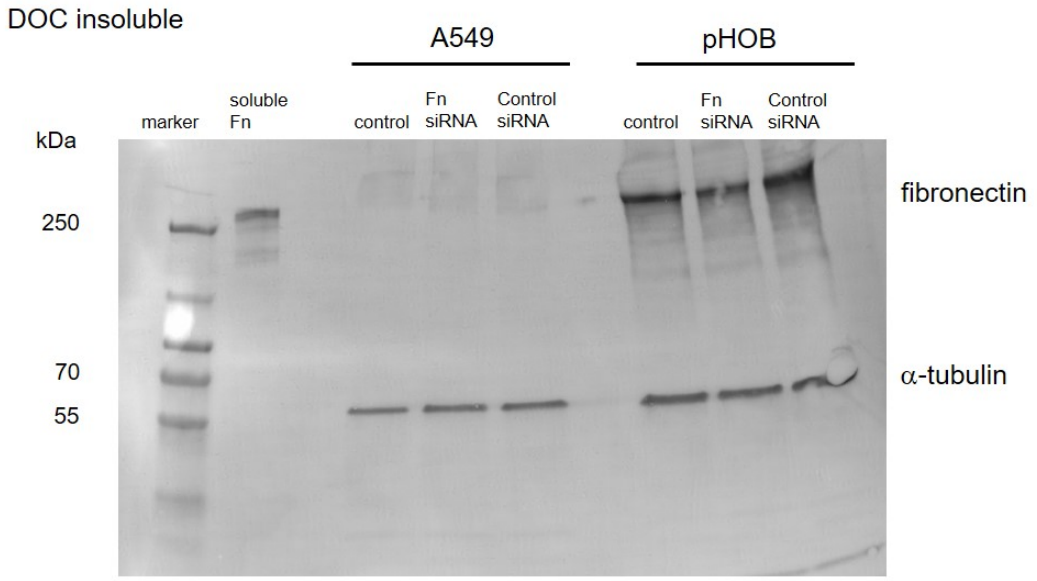


**B**

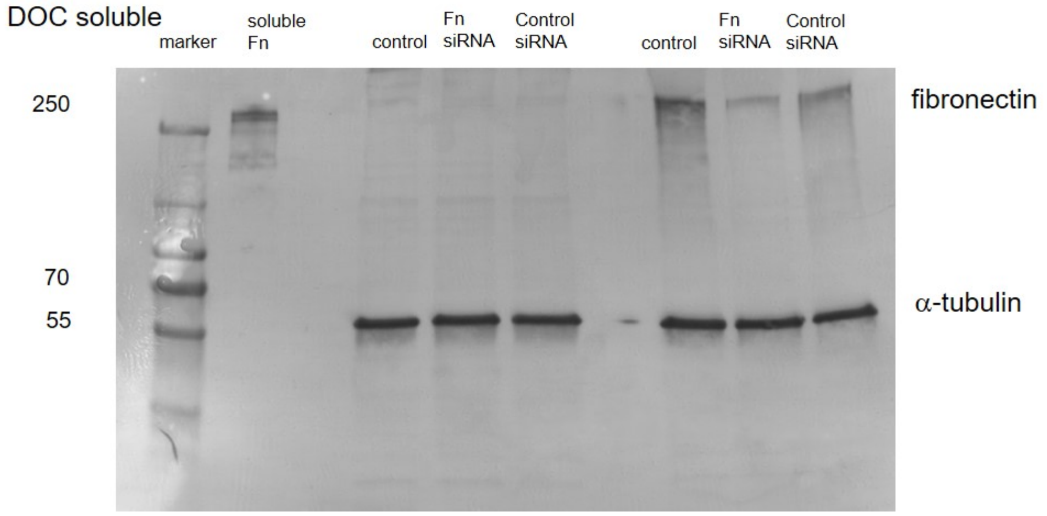


**Fig. S6: Representative unprocessed Western blots of DOC solubility assay of homogenates of pHOB and A549 after siRNA treatment (see also Fig. S5).** Cells were grown for three days and incubated with Fn siRNA or control siRNA for two more days. In the lane labeled "soluble Fn", soluble Fn was applied, which was used as control. **(A)** A549 and pHOB, DOC insoluble fraction **(B)** A549 and pHOB, DOC soluble fraction.
