# Supplementary material for: More Is Not Always Better—the Double-Headed Role of Fibronectin in Staphylococcus aureus Host Cell Invasion
Source: mBio. 2021 Oct 19;12(5):e01062-21. doi: 10.1128/mBio.01062-21 (PMC8524341; doi:10.1128/mBio.01062-21)
Supplement: FIG S7 [file mbio.01062-21-sf007.pdf]

Fig. S7

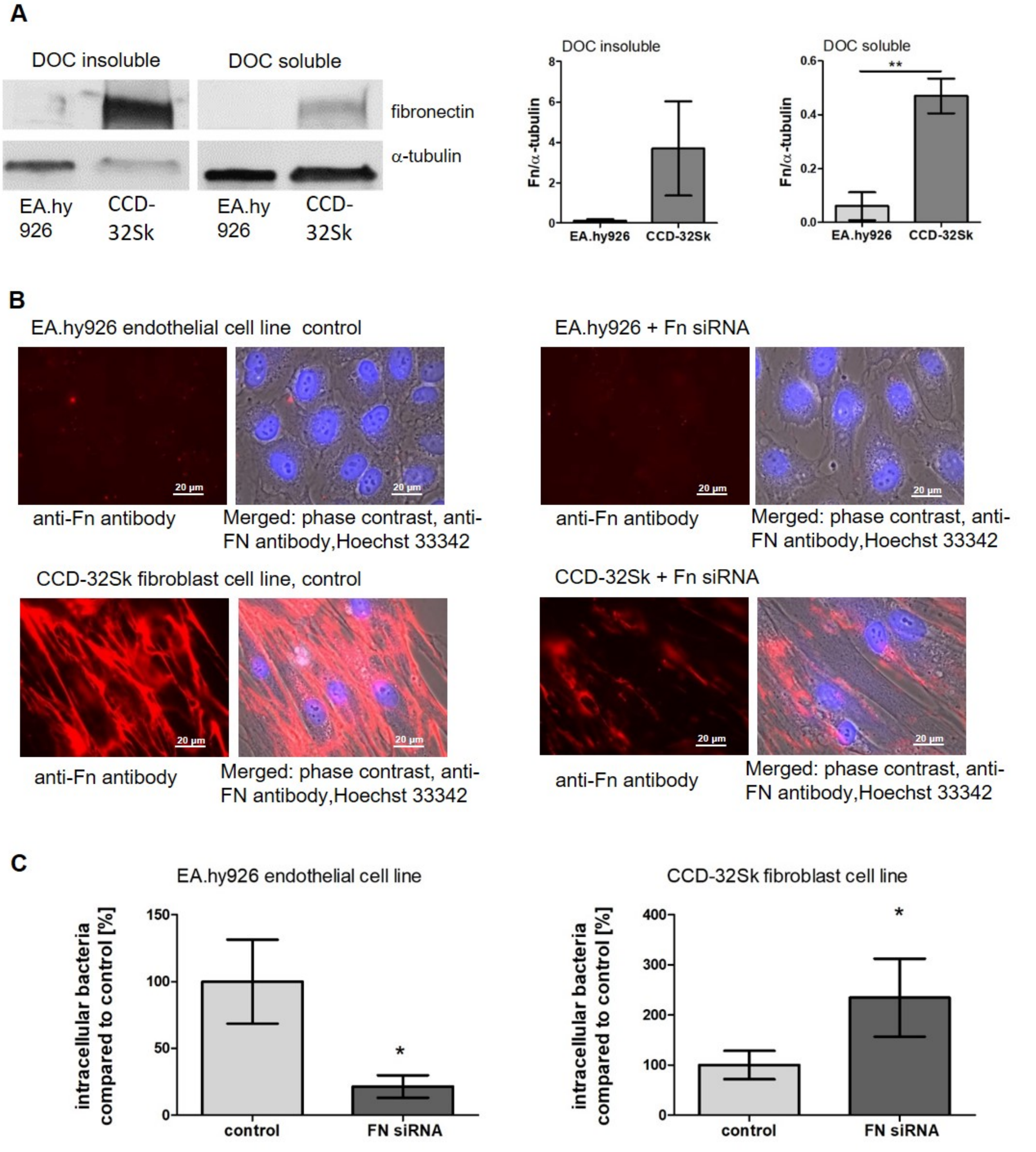

Fig. S7: Augmenting effect on bacterial uptake efficacy by Fn silencing in fibroblast cell line. **(A)** After three days of growth, the DOC insoluble and the DOC soluble protein fraction of EA.hy926 endothelial cell line and CCD-32Sk fibroblast cell line were harvested and analyzed by Western blotting (see also Fig. S2). Data are means ± SD of Western blot quantification of three independent experiments. ** $p \leq 0.01$, unpaired t-test. **(B)** Representative images of Fn expression on EA.hy926 endothelial cells and CCD-32Sk fibroblasts without and with Fn silencing by Fn siRNA detected by immunofluorescence microscopy using an antibody against Fn (red) and Hoechst 33342 for nucleic acid staining (blue). **(C)** Lysostaphin protection assay was performed to quantify intracellular bacteria one hour post infection of either EA.hy926 or CCD-32Sk cells with *S. carnosus* TM300(pFNBA4) without and with Fn silencing by Fn siRNA. Data are means ± SD of three independent experiments. * $p<0.05$, unpaired t-test.
