# Supplementary material for: More Is Not Always Better—the Double-Headed Role of Fibronectin in Staphylococcus aureus Host Cell Invasion
Source: mBio. 2021 Oct 19;12(5):e01062-21. doi: 10.1128/mBio.01062-21 (PMC8524341; doi:10.1128/mBio.01062-21)
Supplement: FIG S8 [file mbio.01062-21-sf008.pdf]

Fig. S8

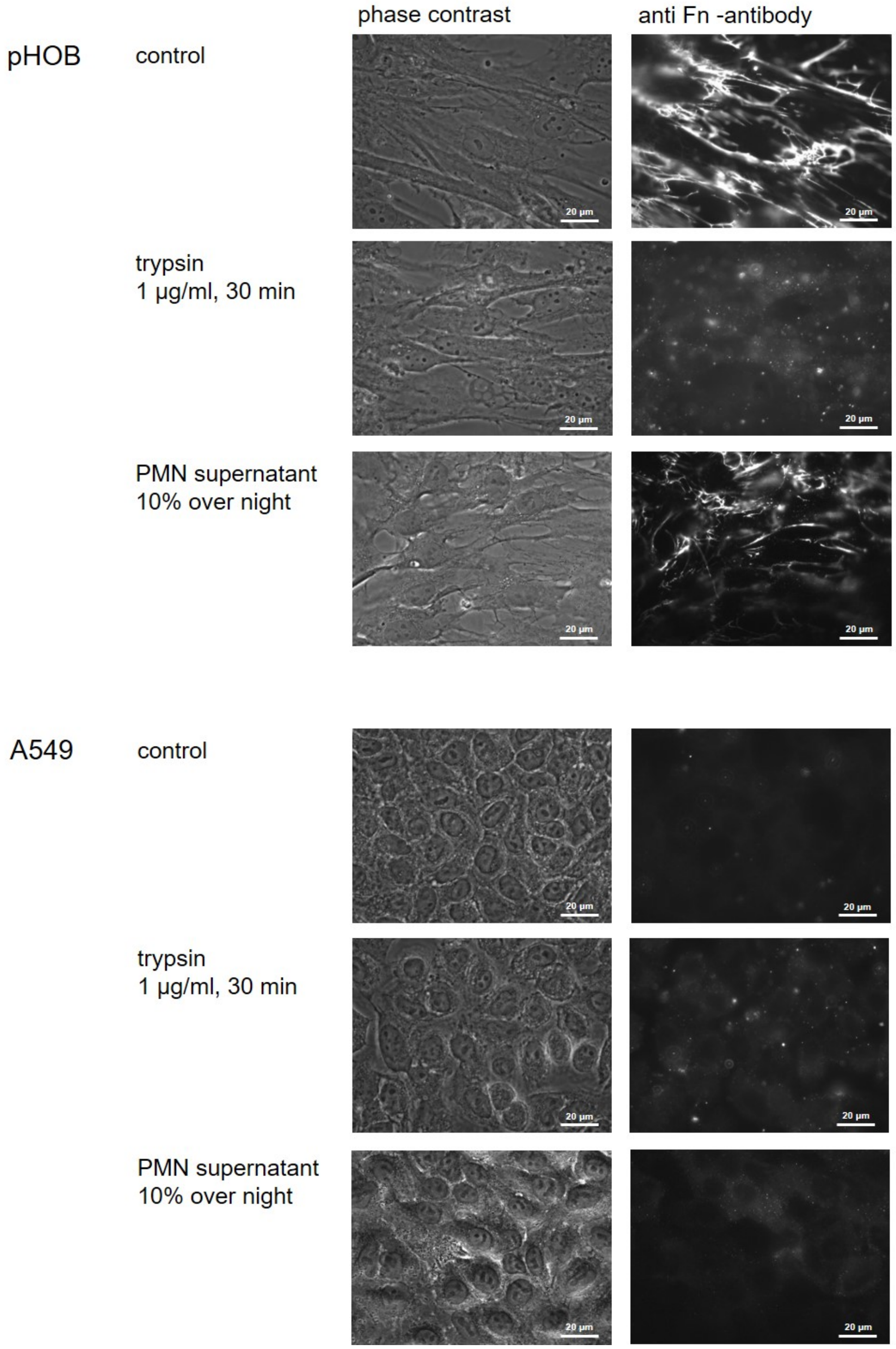

Fig. S8: Fn fibrils are destroyed by trypsin and supernatant of lysed neutrophils. Representative images of phase contrast and immunofluorescence microscopy. pHOB and A549 were grown for two days in normal growth medium supplemented with FBS and then treated either with 1 µg/ml trypsin for 30 min in invasion medium or over night with 10 % supernatant of lysates of polymorphonuclear neutrophils (PMN, destroyed by ultrasound) in basal medium supplemented with 0.21% BSA. Cells were fixed and stained for Fn.
